# Harnessing Real-World Data to Inform Decision-Making: Multiple Sclerosis Partners Advancing Technology and Health Solutions (MS PATHS)

**DOI:** 10.3389/fneur.2020.00632

**Published:** 2020-08-07

**Authors:** Ellen M. Mowry, Robert A. Bermel, James R. Williams, Tammie L. S. Benzinger, Carl de Moor, Elizabeth Fisher, Carrie M. Hersh, Megan H. Hyland, Izlem Izbudak, Stephen E. Jones, Bernd C. Kieseier, Hagen H. Kitzler, Lauren Krupp, Yvonne W. Lui, Xavier Montalban, Robert T. Naismith, Jacqueline A. Nicholas, Fabio Pellegrini, Alex Rovira, Maximilian Schulze, Björn Tackenberg, Mar Tintore, Madalina E. Tivarus, Tjalf Ziemssen, Richard A. Rudick

**Affiliations:** ^1^Johns Hopkins University, Baltimore, MD, United States; ^2^Cleveland Clinic, Cleveland, OH, United States; ^3^Biogen, Cambridge, MA, United States; ^4^Washington University in St. Louis, St. Louis, MO, United States; ^5^Cleveland Clinic Lou Ruvo Center for Brain Health, Las Vegas, NV, United States; ^6^University of Rochester Medical Center, Rochester, NY, United States; ^7^Center of Clinical Neuroscience, University Clinic Carl Gustav Carus, TU Dresden, Dresden, Germany; ^8^New York University, New York, NY, United States; ^9^Vall d'Hebron University Hospital, Barcelona, Spain; ^10^OhioHealth, Columbus, OH, United States; ^11^University Hospital of Giessen and Marburg, Marburg, Germany

**Keywords:** learning health system, multiple sclerosis, MS PATHS, digital health technology, standardized brain magnetic resonance imaging

## Abstract

**Background:** Multiple Sclerosis Partners Advancing Technology and Health Solutions (MS PATHS) is the first example of a learning health system in multiple sclerosis (MS). This paper describes the initial implementation of MS PATHS and initial patient characteristics.

**Methods:** MS PATHS is an ongoing initiative conducted in 10 healthcare institutions in three countries, each contributing standardized information acquired during routine care. Institutional participation required the following: active MS patient census of ≥500, at least one Siemens 3T magnetic resonance imaging scanner, and willingness to standardize patient assessments, share standardized data for research, and offer universal enrolment to capture a representative sample. The eligible participants have diagnosis of MS, including clinically isolated syndrome, and consent for sharing pseudonymized data for research. MS PATHS incorporates a self-administered patient assessment tool, the Multiple Sclerosis Performance Test, to collect a structured history, patient-reported outcomes, and quantitative testing of cognition, vision, dexterity, and walking speed. Brain magnetic resonance imaging is acquired using standardized acquisition sequences on Siemens 3T scanners. Quantitative measures of brain volume and lesion load are obtained. Using a separate consent, the patients contribute DNA, RNA, and serum for future research. The clinicians retain complete autonomy in using MS PATHS data in patient care. A shared governance model ensures transparent data and sample access for research.

**Results:** As of August 5, 2019, MS PATHS enrolment included participants (*n* = 16,568) with broad ranges of disease subtypes, duration, and severity. Overall, 14,643 (88.4%) participants contributed data at one or more time points. The average patient contributed 15.6 person-months of follow-up (95% CI: 15.5–15.8); overall, 166,158 person-months of follow-up have been accumulated. Those with relapsing–remitting MS demonstrated more demographic heterogeneity than the participants in six randomized phase 3 MS treatment trials. Across sites, a significant variation was observed in the follow-up frequency and the patterns of disease-modifying therapy use.

**Conclusions:** Through digital health technology, it is feasible to collect standardized, quantitative, and interpretable data from each patient in busy MS practices, facilitating the merger of research and patient care. This approach holds promise for data-driven clinical decisions and accelerated systematic learning.

## Introduction

The multiple sclerosis (MS) treatment landscape has experienced a dramatic evolution over the past two decades; there are now 16 approved disease-modifying therapies (DMTs). Approaches to personalized medicine in MS, however, have not kept pace. Defining disease prognosis, treatment outcomes, monitoring treatment response, and determining optimal treatment sequencing remain variable and somewhat subjective in MS practice. Personalized medicine efforts have focused both on identifying informative patient phenotypes (1) and effectively integrating and visualizing individual patient data (2).

The rigorous collection and analysis of real-world data may accelerate the development of personalized medicine in MS and address some of these gaps (3–5). Opportunities and challenges related to data pooling and data standardization in MS were recognized by early pioneers, beginning decades ago (6, 7), and recent progress has been summarized (8, 9). These efforts have ushered in an era of data standardization and pooling in an attempt to extend systematic learning beyond structured research protocols to more representative real-world populations. For maximum impact, data should include standardized and quantitative clinical, radiologic, and biological phenotyping from a heterogeneous population representative of the diversity of patients with MS seen in everyday clinical practice.

The learning health system (LHS) model, as proposed by the Institute of Medicine, outlines a method to enable broad-scale quantitative patient phenotyping through the merging of clinical research and healthcare delivery (4, 10). The LHS (also known as evidence-generating medicine) seeks to produce better outcomes and research based on real-time data acquisition (10). The tenets of the LHS concept include collection of standardized, meaningful data on every patient seeking care, increased engagement of patients in the process of care, use of quantitative data for clinical decision-making, and aggregation of data from populations for systematic learning (10). The LHS represents a culture of continuous learning, feeding insights back into care delivery to continuously reanalyze, revalidate, and improve outcomes.

The current report describes the first LHS in MS, Multiple Sclerosis Partners Advancing Technology and Health Solutions (MS PATHS). In MS PATHS, quantitative clinical and imaging data are collected in a standardized manner for each patient as part of routine care. Data are collected as part of the patient's clinical evaluation, while—with a patient's permission—the data are pseudonymized and aggregated for systematic learning. The program leverages technology and patient engagement to automate data collection and analysis, minimizing the burden on the care system and providers. The specific goals of MS PATHS are to better understand the disease, identify the predictors of therapeutic responses, define and measure outcomes during the course of care, and develop approaches to personalized medicine.

We aim to demonstrate the feasibility of implementing the key features of an LHS in MS by describing the patient population enrolled in MS PATHS to date, initial data completion rates, differences between MS PATHS and typical MS clinical trial patients, heterogeneity of practice across the network, and utilization of the research data.

## Materials and Methods

### Initial Design

MS PATHS is a collaborative network of healthcare institutions that have standardized elements of their clinical assessments and collaborated with Biogen to implement a centralized database for research purposes. This network was designed based on the guiding principles described in Table 1. In 2014, a group of stakeholders from Cleveland Clinic, Johns Hopkins University, New York University, and Biogen began the planning process to best design the tools, systems, and governance needed for an LHS in MS.

**Table 1:** Guiding principles for MS PATHS.

Engage all healthcare providers and nearly all patients with MS in a healthcare institution
Standardize, quantify, and maximize data collected as part of standard of care•Identify key data points needed to interpret test results, achieve practice standards/meaningful use, and enable the generation of new clinical knowledge
Leverage technology to enable data collection in clinical practice • Make it possible to collect data on all participating patients, which is too time consuming using traditional research methods • Leverage patient-reported data to the greatest extent possible • Make MSPT data available at the point of care and simultaneously aggregate data for learning Collect data outside of standard of care *via* separately consented substudiesEnsure transparent governance by multi-stakeholder groupBecome recognized as meaningful by patients, providers, payers, and other stakeholders

*MS, multiple sclerosis; MS PATHS, Multiple Sclerosis Partners Advancing Technology and Health Solutions; MSPT, Multiple Sclerosis Performance Test*.

Methods for standardized clinical and imaging data collection that do not increase the burden on providers or generate significant incremental cost were an important aspect of the planning process. Technology was developed to allow patient self-administered clinical assessment, resulting in standardized, high-quality clinical data. Technician-based testing—specifically, Multiple Sclerosis Functional Composite testing—was adapted to a series of patient self-administered iPad-based tests to provide quantitative data related to processing speed, low-contrast visual acuity, manual dexterity, and walking speed (11). This was done in order to facilitate the neuroperformance testing for every patient with MS, even in a busy clinical practice (12–14). The iPad-based clinical assessment tool, called the Multiple Sclerosis Performance Test (MSPT), also enabled the collection of a structured patient history.

Because the clinical assessment was patient self-administered, there are differences between the MSPT and the physician-derived measures used in clinical trials and traditional MS practice registries. Neurologist-determined relapses and Expanded Disability Status Scale were replaced by patient-reported relapse and Patient Determined Disease Steps. Prior studies support the validity of self-reported relapses (15, 16) and showed a strong correlation between Patient Determined Disease Steps and Expanded Disability Status Scale (17–19). The MSPT also enabled the collection of quality-of-life data. The Quality of Life in Neurological Disorders (Neuro-QoL) measure was selected as a standardized, well-validated patient-reported outcome instrument (20–22). The computer-adapted version of Neuro-QoL was incorporated into the MSPT to minimize administration time.

Two standardized magnetic resonance imaging (MRI) acquisition sequences were incorporated into routine MS imaging protocols to enable the reliable calculation of brain atrophy and lesions at the point of care. Through a collaboration with Siemens Healthineers, a software prototype is currently in development, with a focus on workflow integration and performance adequate for individual patient clinical decision-making.

To enable translational research, an MS PATHS research substudy was implemented to enable the collection of blood samples under a research protocol that could be linked to the standardized clinical and imaging data for future analyses.

### Participating Healthcare Institutions

To be eligible for MS PATHS, the participating healthcare institutions had to have an MS center with an active census of ≥500 patients that routinely used functional measures and MRI in their clinical practice and a willingness to further standardize aspects of their clinical and radiological assessments. In addition, each participating healthcare institution had to have at least one Siemens 3T MRI scanner available for clinical use. Each participating center had to be willing to implement a centralized health information exchange architecture to transfer the data to a research database and obtain approval of the project protocol by the institutional review board or ethics committee, information technology (IT) security, and/or data privacy committee and agree to adhere to Good Clinical Practice and ethical principles as outlined in the Declaration of Helsinki. The participating healthcare institutions of each investigator receive financial compensation for data shared and biosamples collected as part of this program based on fair market value.

### Patients

Patients with a confirmed diagnosis of MS, including clinically isolated syndrome, and the ability to understand the purpose and the risks of the project are eligible to enroll in MS PATHS. In contrast with a traditional prospective observational study, the sites in MS PATHS agree to adopt the outlined standard of care, and the patients provide authorization for the use of protected health information in accordance with national and local subject privacy regulations. Authorization format was determined by the local institutional review board or ethics committee and ranges from the use of a standard medical information privacy waiver (four institutions) to an oral consent (one institution) to a full informed consent (five institutions). The investigators and research coordinators are encouraged to invite all patients at each MS center to participate. At steady state, the network aims to have 80% of the MS patients at each participating MS center enrolled in MS PATHS.

### Procedures

Upon enrollment, the patients are assigned a unique MS PATHS identification number that acts as the patient identifier in the LHS. This allows linkage of pseudonymized data from different sources for the same patient. The authorization form allows for prospective data sharing as well as sharing of data from 12 months before the date of consent.

The data elements collected routinely for patients in MS PATHS are listed in Table 2. Clinical data are collected using the MSPT. In addition, structured clinical data are shared from electronic medical records at each institution. Imaging data include brain MRIs acquired using two standardized sequences (three-dimensional fluid-attenuated inversion recovery and three-dimensional T1 acquired on Siemens 3T scanners; Table 3), information from radiology reports, and quantitative measures of brain atrophy and lesion metrics derived from an image analysis software prototype.

**Table 2:** Current data elements collected in the MS PATHS learning health system.

Patient demographic information (MSPT and EMR) • Age (EMR) • Gender (MSPT) • Race (MSPT) • Ethnicity (MSPT) • Education (MSPT) • Employment status (MSPT) • Insurance coverage type (MSPT and EMR) • Living situation (MSPT) • Employment status (MSPT) • Dominant hand (MSPT)
MS and other medical history (MSPT and EMR) • MS subtype (MSPT) • Age at first MS symptom onset (MSPT) • Age at MS diagnosis (MSPT) • Mobility aid use (MSPT) • Smoking status (EMR)
Physical and laboratory assessments (EMR) • Weight and height • Body mass index • Blood pressure • Laboratory test values
Medications (MSPT and EMR) • Patient self-reported use of MS disease-modifying therapy (MSPT) • Medication list (EMR)
Patient-reported outcomes and tests (MSPT) • Patient-reported relapses • Patient Determined Disease Steps • Neuro-QoL ° Mental ▪ Ability to participate in social roles and activities ▪ Anxiety ▪ Cognition ▪ Depression ▪ Emotional and behavioral dyscontrol ▪ Positive affect and well-being ▪ Satisfaction with social roles and activities ▪ Stigma ° Physical ▪ Fatigue ▪ Lower extremity function (mobility) ▪ Sleep disturbance ▪ Upper extremity function (fine motor, ADL) • Neuroperformance testing ° Walking Speed Test ° Processing speed test ° Manual dexterity test ° Contrast sensitivity test
MRI-related data • 3D T1 and 3D FLAIR MRI • Radiologist report of number of new or enlarging T2 lesions (EMR) • Radiologist report of number of enhancing T2 lesions (EMR) • Quantitative brain volume metrics^*^ • Quantitative T2 lesion metrics^*^

*3D, three-dimensional; ADL, activities of daily living; EMR, electronic medical record; FLAIR, fluid-attenuated inversion recovery; MRI, magnetic resonance imaging; MS, multiple sclerosis; MSPT, Multiple Sclerosis Performance Test; Neuro-QoL, Quality of Life in Neurological Disorders*.

* *Under development*.

**Table 3:** Standardized Siemens 3T brain MRI sequence parameters for MS PATHS.

**Parameter**	**3D FLAIR MS-Pie (SPACE)**	**3D T1 MS-Pie (MPRAGE)**
Resolution (mm)	1 × 1 × 1	1 × 1 × 1
Field of view	256 × 256 × 176	256 × 256 × 176
Orientation	Sagittal	Sagittal
Total acquisition time (min:s)	6:27	5:12
Repetition time (ms)	5,000	2,300
Echo time (ms)	392	2.96
Inversion time (ms)	1,800	900

*3D, three-dimensional; FLAIR, fluid-attenuated inversion recovery; MPRAGE, magnetization-prepared rapid gradient-echo imaging; MRI, magnetic resonance imaging; ms, milliseconds; MS PATHS, Multiple Sclerosis Partners Advancing Technology and Health Solutions*.

Data collected during patient care visits can also be linked to substudies. Each substudy requires a separate protocol and informed consent to allow for research assessments or procedures and for the linkage of the substudy data with patient care data. An example is an ongoing biorepository substudy that collects a one-time blood sample for future genomic analyses (10 ml for adult patients and 6 ml for pediatric patients) and repeated blood samples for future biomarker analyses (33 ml for adult patients and 13.5 ml for pediatric patients) not more frequently than every 6 months. The clinical phenotyping information for any genetic or biomarker analyses will be derived from the routine clinical care data that are shared as part of MS PATHS.

### Governance

The governance for MS PATHS requires a structure and a process that:

1. Are fair, transparent, and compliant for all participating organizations;
2. Assure confidence from all participating organizations;
3. Foster collaboration;
4. Reinforce the clinical and research integrity of participating healthcare institutions;
5. Ensure that all participating organizations have sufficient freedom to operate; and
6. Foster innovation, limit risk, and enable long-term success.

A steering committee was tasked with:

1. Providing strategic and operational guidance;
2. Setting the MS PATHS scientific strategy;
3. Creating and overseeing data and sample access rules;
4. Monitoring performance; and
5. Providing other oversight activities as needed.

The steering committee consists of six representatives from participating healthcare institutions and one representative from Biogen. Three steering committee seats are reserved for investigators from the institutions that collaborated with Biogen on the initial planning process (Cleveland Clinic Foundation, Johns Hopkins University, and New York University), and one seat each is designated for an additional US healthcare institution, an EU healthcare institution, and a radiologist in MS PATHS.

The steering committee governs a set of data and sample access subcommittees responsible for approving requests for MS PATHS data and samples. The healthcare institutions and Biogen each have separate data and sample access committees. Biogen employees do not sit on the healthcare institution committees and *vice versa*. Separate subcommittees for Biogen and participating healthcare institutions were set up to ensure the independence of research led by the participating healthcare institutions. The healthcare institution and Biogen subcommittees operate under the same procedures, and all approved uses of the data or the samples are posted on the MS PATHS research website, which is accessible to Biogen and the participating healthcare institutions to ensure transparency and promote collaboration. In addition to approving data or sample requests, these subcommittees are also responsible for reviewing resulting presentations or publications to ensure alignment with the original request. Biogen, as the sponsor, has no role in writing or editing publications unless a Biogen employee is a co-author.

### Standardization

Participating healthcare institutions have implemented the MSPT (11, 13, 14), an iPad-based medical assessment tool that quantifies major MS-associated motor, visual, and cognitive symptoms and quality-of-life outcomes. The MSPT incorporates a structured patient history (gathers the patient's relevant demographic and socioeconomic information, MS history, MS treatment information, and self-reported disability using the Patient Determined Disease Steps) (17–19), 12 subscales of the Neuro-QoL (20–22), and an electronic adaptation of the Multiple Sclerosis Functional Composite (23). The adapted Multiple Sclerosis Functional Composite includes a processing speed test that is similar to the Symbol Digit Modalities Test; a manual dexterity test, similar to the 9-Hole Peg Test; a contrast sensitivity test, similar to the Sloan low-contrast visual acuity test; and a 25-foot walking speed test, similar to the Timed 25-Foot Walk. The MSPT is administered during routine clinical visits, typically prior to meeting with the healthcare provider. Depending on the institution, the results are immediately available to the healthcare provider *via* the patient's electronic medical record or *via* a results screen on the MSPT. The processing speed test, manual dexterity test, contrast sensitivity test, and 25-foot walking speed test demonstrate reliability, validity, and sensitivity to MS outcomes (13, 14).

In MS PATHS, the participating healthcare institutions collaborate with Biogen and Siemens Healthineers to implement two highly standardized MRI acquisition sequences (a three-dimensional magnetization-prepared rapid gradient-echo imaging and three-dimensional fluid-attenuated inversion recovery) that are readily available product sequences and consistent with recent MS imaging guidelines (24). The participating healthcare institutions have also implemented standardized fields in the radiology report for assessment of new or enlarging T2 lesions and contrast-enhancing lesions (if applicable).

### Health Information Exchange Architecture

MS PATHS is enabled by health IT that supports the secure transfer, processing, and harmonization of clinical data for research purposes. Similar to the hub-and-spoke model of data transfer in health information exchanges, clinical source systems such as the electronic medical record systems at participating healthcare institutions (the spokes) will send data through a series of intermediary systems before the data are passed through to the central MS PATHS research database (also known as the LHS).

Each participating institution is supported by a separate gateway that is responsible for applying consent logic to incoming data and then conducting any needed format transformations to enable ingestion by a central data broker. The two data brokers, one for the United States and one for the European Union, manage a patient registration and consent index as well as a de-identification tool that pseudonymizes data. Patient data other than consent and registration information are deleted from each broker after five business days.

Biogen has contracted with an IT vendor to act as a trusted third party to build and operate the MS PATHS gateways and data brokers. This IT vendor is responsible for protecting and processing identifiable patient data before they are pseudonymized and sent to the LHS.

In the United States, the vendor has entered into business associate agreements with each participating healthcare institution. Biogen does not have access to the data in either broker. In the European Union, each healthcare institution has contracted with an additional IT vendor to serve as the initial intermediary prior to the edge gateway. The EU IT vendor completes an initial pseudonymization of the data before they are transferred to an institution's gateway and subsequently to the EU broker. Once the data are pseudonymized in a broker, they are transferred to the LHS. The LHS is logically isolated from the brokers so that both Biogen and researchers from participating healthcare institutions do not have access to identifiable patient data.

In the LHS, data are harmonized using industry-accepted clinical terminology standards such as SNOMED, Logical Observation Identifiers Names and Codes, RxNorm, National Drug Code, and International Statistical Classification of Diseases and Related Health Problems. The LHS data are available to be requested for research purposes by any researcher at a participating healthcare institution or at Biogen.

### Statistical Analysis

Descriptive analyses were used to describe MS PATHS patient characteristics at the time of a patient's initial MSPT assessment. Continuous variables were reported as mean (SD) and categorical variables as percentages. Between-group differences were assessed using *t* tests and chi-square (χ^2^) tests, as appropriate.

To test whether MS PATHS is more diverse than typical phase 3 clinical trials in MS, the characteristics of patients with relapsing–remitting MS (RRMS) enrolled in MS PATHS were compared with patients with RRMS pooled from six phase 3 randomized controlled clinical trials (RCTs) sponsored by Biogen (25–29). Comparisons of patients from MS PATHS vs. the pooled RCTs were made using Wilcoxon rank-sum tests and Pearson χ^2^ tests. Standardized mean or proportion differences (i.e., effect sizes) were also calculated. C statistics for membership (30) and predicted probability (i.e., propensity score) distributions were estimated using multivariable logistic regression models to assess overall baseline characteristics (i.e., case–mix) similarity. To assess the extent of overlap in the two populations (MS PATHS vs. RCTs), 1:1 propensity score matching (31) was performed based on a 5:1 greedy match algorithm (32). Comparisons between the matched sample characteristics were made using McNemar's test or Wilcoxon signed rank test. Effect sizes and C statistics for membership were also reported.

To assess heterogeneity in terms of assessment frequency, separate Kaplan–Meier plots were created for each center, describing the time (months) between assessments. Heterogeneity at the level of the healthcare institution was tested using log-rank tests. Separate analyses were conducted for MSPT and MRI assessments.

## Results

### MS PATHS Patient Population

As of August 5, 2019, 16,568 patients from 10 participating institutions in the United States (*n* = 7) and the European Union (*n* = 3) agreed to share their clinical data, representing 71.4% of patient census seen in the MS clinics within the network. As of the cutoff date for the data included in this manuscript, the number of withdrawals was 158 (0.95%) and ranged from 0.12 to 2.91% of participants across the sites.

Of 16,568 enrolled patients, 14,643 patients completed at least one MSPT assessment; the characteristics at the time of initial MSPT assessment for these patients are shown in Table 4. Mean (SD) age was 47.0 (12.4) years and the population was largely female [*n* = 10,712 (73.2%)] and predominantly white, although the absolute number of non-white participants is substantial. Mean (SD) Neuro-QoL T-scores ranged from 45.4 (9.6) to 52.7 (10.2).

**Table 4:** Patient demographic and clinical characteristics at initial assessment.

Characteristic^*^	**Value**
Mean (SD) age (years)^†^	47.0 (12.4)
Female, *n* (%)^‡^	10,712 (73.2)
Race/area of origin, *n* (%)^‡^	
United States—race	11,236 (76.7)
White	8,933 (79.5)
Black or African American	1,419 (12.6)
Asian	75 (0.7)
American Indian or Alaska Native	46 (0.4)
Native Hawaiian or other Pacific Islander	10 (0.1)
Multiple	290 (2.6)
Other/unknown	368 (3.3)
Choose not to report	95 (0.9)
European Union—area of origin	3,407 (23.3)
Western Europe	2,927 (85.9)
Eastern Europe	197 (5.8)
Asia	12 (0.4)
Multiple	64 (1.9)
Other/unknown	138 (4.1)
Choose not to report	69 (2.0)
Age at diagnosis (years)^§^	35.4 (11.2)
Age at first symptoms (years)^¶^	32.6 (11.4)
MS subtype, *n* (%)^‡^	
Relapsing remitting	8,708 (59.5)
Secondary progressive	2,504 (17.1)
Progressive relapsing	1,247 (8.5)
Primary progressive	1,100 (7.5)
Missing	1,084 (7.4)
Number of relapses in past 12 months, *n* (%)^‡^	
0	7,615 (52.0)
1	3,156 (21.6)
2	1,915 (13.1)
≥3	1,705 (11.6)
Missing	252 (1.7)
Baseline DMT use^‡^	
Dimethyl fumarate	1,966 (13.4)
Glatiramer acetate	1,761 (12.0)
Fingolimod	1,645 (11.2)
Interferon^#^	1,492 (10.2)
Natalizumab	1,384 (9.5)
Ocrelizumab	748 (5.1)
Teriflunomide	562 (3.8)
Rituximab	272 (1.9)
Alemtuzumab	230 (1.6)
Other	115 (0.8)
Not taking any medication/medication not listed	4,418 (30.2)
Missing	50 (0.3)
Neuro-QoL *T*-score^**^	
Mental
Ability to participate in social roles	47.3 (8.0)
Anxiety	51.1 (9.4)
Cognitive functioning	46.2 (9.0)
Depression	47.3 (8.0)
Emotional behavioral dyscontrol^†^	50.6 (9.9)
Positive affect or well-being^†^	52.7 (7.2)
Satisfaction with social roles	47.0 (7.5)
Stigma	47.9 (8.5)
Physical
Fatigue	49.4 (9.9)
Lower extremity function	46.7 (11.2)
Sleep	52.7 (10.2)
Upper extremity function	45.4 (9.6)

*DMT, disease-modifying therapy; MS, multiple sclerosis; Neuro-QoL, Quality of Life in Neurological Disorders*.

* *Data are reported as mean (SD) or n (%) for continuous and categorical variables, respectively*.

† *n = 14,484*,

†† *n = 11,567*,

‡ *n = 14,643*,

§ *n = 13,904*,

¶ *n = 14,236*.

# *Includes interferon beta-1a, interferon beta-1b, interferon beta-other, and peginterferon beta-1a*.

** *Mean (SD) score reference value 50 (10); n = 11,827*.

At their initial MSPT assessment, 69.6% of 14,643 patients reported the use of a DMT. The most frequently reported DMTs were dimethyl fumarate, glatiramer acetate, and fingolimod. DMT use was highest in patients with RRMS [6,495 (74.6%)] compared with patients with progressive MS [1,662 (66.4%) secondary progressive, 540 (49.1%) primary progressive, and 806 (64.6%) progressive relapsing; χ^2^ = 349.5; *p* < 0.0001].

The neuroperformance scores at initial assessment are summarized in Table 5. Patients with RRMS performed better on neuroperformance testing compared with patients with progressive disease.

**Table 5:** Neuroperformance scores at initial assessment.

**Mean (SD) parameter**	**All**	**RRMS**	**SPMS**	**PRMS**	**PPMS**	**F test, *p* value**
Processing speed test (number correct)	46.6 (13.1) *n* = 13,250	50.0 (12.2) *n* = 8,059	41.3 (11.8) *n* = 2,188	40.3 (13.1) *n* = 1,075	39.8 (12.6) *n* = 966	*F* = 532.97 *p* < 0.0001
Contrast sensitivity test (number correct)	34.2 (12.7) *n* = 8,277	36.3 (11.6) *n* = 5,254	29.9 (13.6) *n* = 1,222	30.8 (13.5) *n* = 611	29.6 (14.5) *n* = 541	*F* = 140.74 *p* < 0.0001
Manual dexterity test (seconds)	27.4 (6.8) *n* = 11,829	25.9 (5.9) *n* = 7,505	30.9 (7.4) *n* = 1,834	30.1 (7.6) *n* = 880	31.0 (7.7) *n* = 758	*F* = 459.18 *p* < 0.0001
Walking speed test (seconds)	7.5 (4.6) *n* = 11,758	6.6 (3.2) *n* = 7,504	9.7 (6.4) *n* = 1,778	8.8 (5.3) *n* = 899	10.1 (7.1) *n* = 747	*F* = 362.83 *p* < 0.0001

*PPMS, primary progressive multiple sclerosis; PRMS, progressive relapsing multiple sclerosis; RRMS, relapsing-remitting multiple sclerosis; SPMS, secondary progressive multiple sclerosis*.

### Assessment Completion Rates and Data Volume

The initial completion rates for the MSPT component modules ranged from 56.5% (8,277/14,643; contrast sensitivity test) to 100.0% (14,643/14,643; MyHealth module; Table 6). Missing data resulted from the coordinator disabling the module for patients unable or unwilling to complete the test or the patient canceling the test themselves. Longitudinal MSPT data were available for 72.7% (10,640/14,643) of patients (Table 7). The average patient contributed 15.6 person-months of follow-up (95% CI: 15.5–15.8); overall, 166,158 person-months of follow-up have been accumulated.

**Table 6:** MSPT module completion rates at initial assessment.

**Module**	Assigned, n^*^	**Completed, *n* (%)**	**Patient declined or unable to complete, *n* (%)**
Processing speed test	14,643	13,250 (90.5)	1,393 (9.5)
Contrast sensitivity test	14,643	8,277 (56.5)	6,366 (43.5)
Manual dexterity test	14,643	11,829 (80.8)	2,814 (19.2)
Walking speed test	14,643	12,152 (83.0)	2,491 (17.0)
Neuro-QoL^†^	14,643	11,827 (80.8)	2,816 (19.2)
MyHealth^‡^	14,643	14,643 (100.0)	

*MSPT, Multiple Sclerosis Performance Test; Neuro-QoL, Quality of Life in Neurological Disorders*.

* *MSPT assessments taken on the same day are combined into a single record*.

† *A computer-adaptive quality-of-life measure included with the MSPT*.

‡ *A structured patient questionnaire that records demographics, health history, use of multiple sclerosis disease-modifying therapy, and multiple sclerosis status*.

**Table 7:** MS PATHS data volume (*N* = 16,568).

**Assessment**	**Total assessments**	**≥1, *n* (%)**	**≥2, *n* (%)**	**≥3, *n* (%)**	**≥4, *n* (%)**
MSPT	41,187	14,643 (100)	10,640 (72.7)	7,038 (48.1)	4,332 (29.6)
Median (IQR) duration since first assessment (days)			448 (282–650)	553 (390–725)	609 (478–763)
Brain MRI	14,414	8,364 (100)	3,822 (45.7)	1,510 (18.1)	470 (5.6)
Median (IQR) duration since first assessment (days)			469 (350–715)	721 (565–834)	859 (721–1004)
Biobanking	10,223	6,581 (100)	2,584 (39.3)	836 (12.7)	196 (3.0)
Median (IQR) duration since first assessment (days)			364 (224–483)	546 (421–637)	658 (609–736)

*IQR, interquartile range; MRI, magnetic resonance imaging; MS PATHS, Multiple Sclerosis Partners Advancing Technology and Health Solutions; MSPT, Multiple Sclerosis Performance Test*.

A total of 14,414 MRI studies were collected from 8,364 unique patients, including 3,822/8,364 (45.7%) patients with longitudinal MRI data (Table 7). Of the 14,414 MRI studies received, 281 (2.0%) were rejected for being incomplete or not acquired using standardized sequence parameters. Of 14,643 patients with at least one MSPT assessment, 7,622 (52.1%) had at least one standardized 3T MRI.

A one-time genetic sample was collected from 6,320 patients. Other blood samples have been collected at a total of 10,223 biobanking visits from 6,581 unique patients, including 2,584/6,581 (39.3%) with a longitudinal sample collected (Table 7). The 10,223 biobanking visits represent 129,986 individual samples received in the central laboratory. A total of 265 (0.2%) samples were rejected after the initial quality control checks.

### Heterogeneity Observed Across Sites

**Figure 1:**
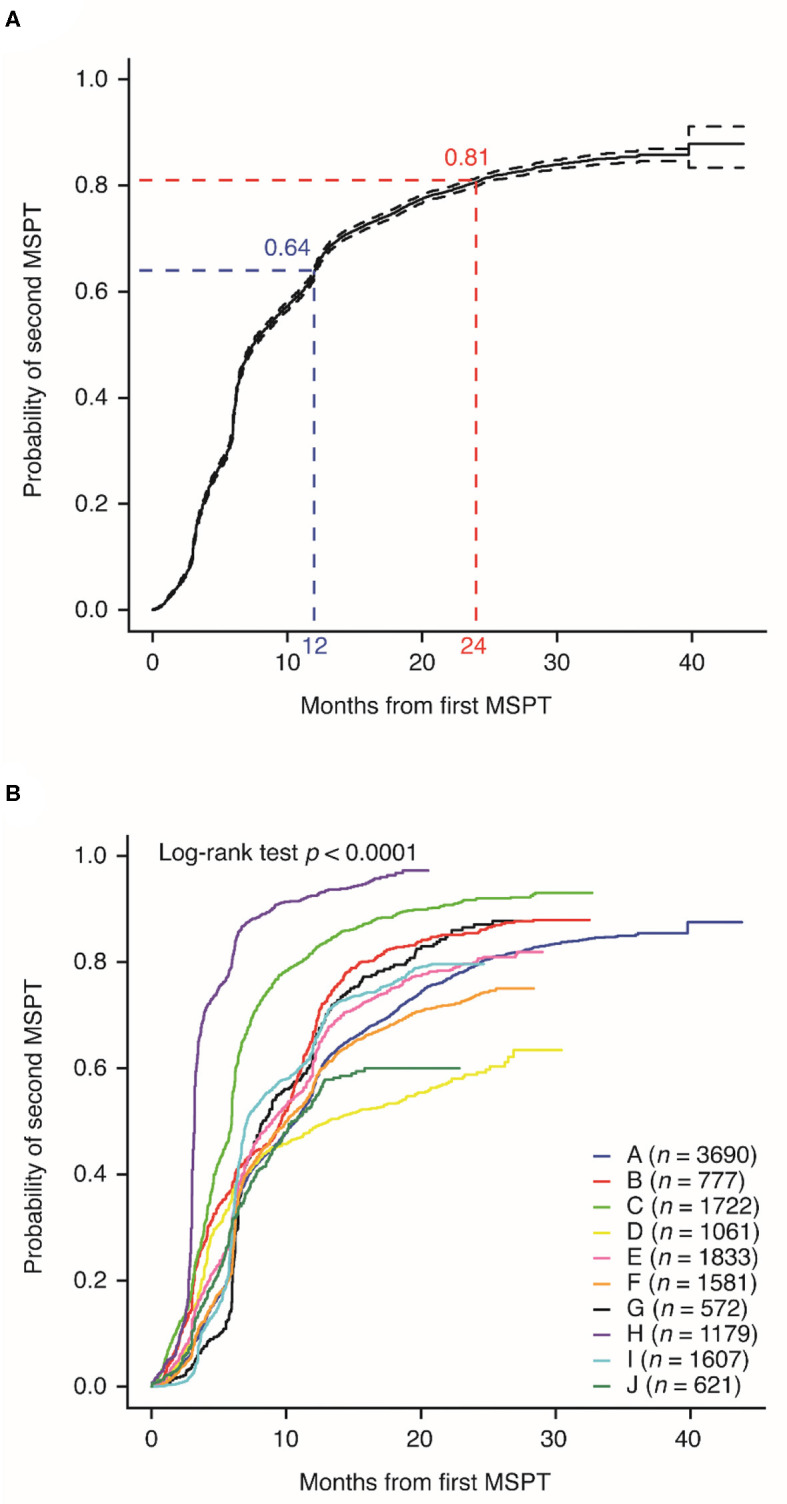
Probability of completing a follow-up Multiple Sclerosis Performance Test (MSPT; as a function of months between the initial MSPT and completing the second MSPT), **(A)** overall and by **(B)** Multiple Sclerosis Partners Advancing Technology and Health Solutions center.

After completing their initial MSPT, 64% and 81% of patients in MS PATHS completed a follow-up MSPT within 12 and 24 months, respectively (Figure 1A). The median time to complete a follow-up MSPT was ~7.4 months after the initial MSPT. The median time to complete a follow-up MSPT varied considerably among the participating healthcare institutions, ranging from 3 to 13 months (Figure 1B; χ^2^ = 2,575; *p* < 0.0001).

**Figure 2:**
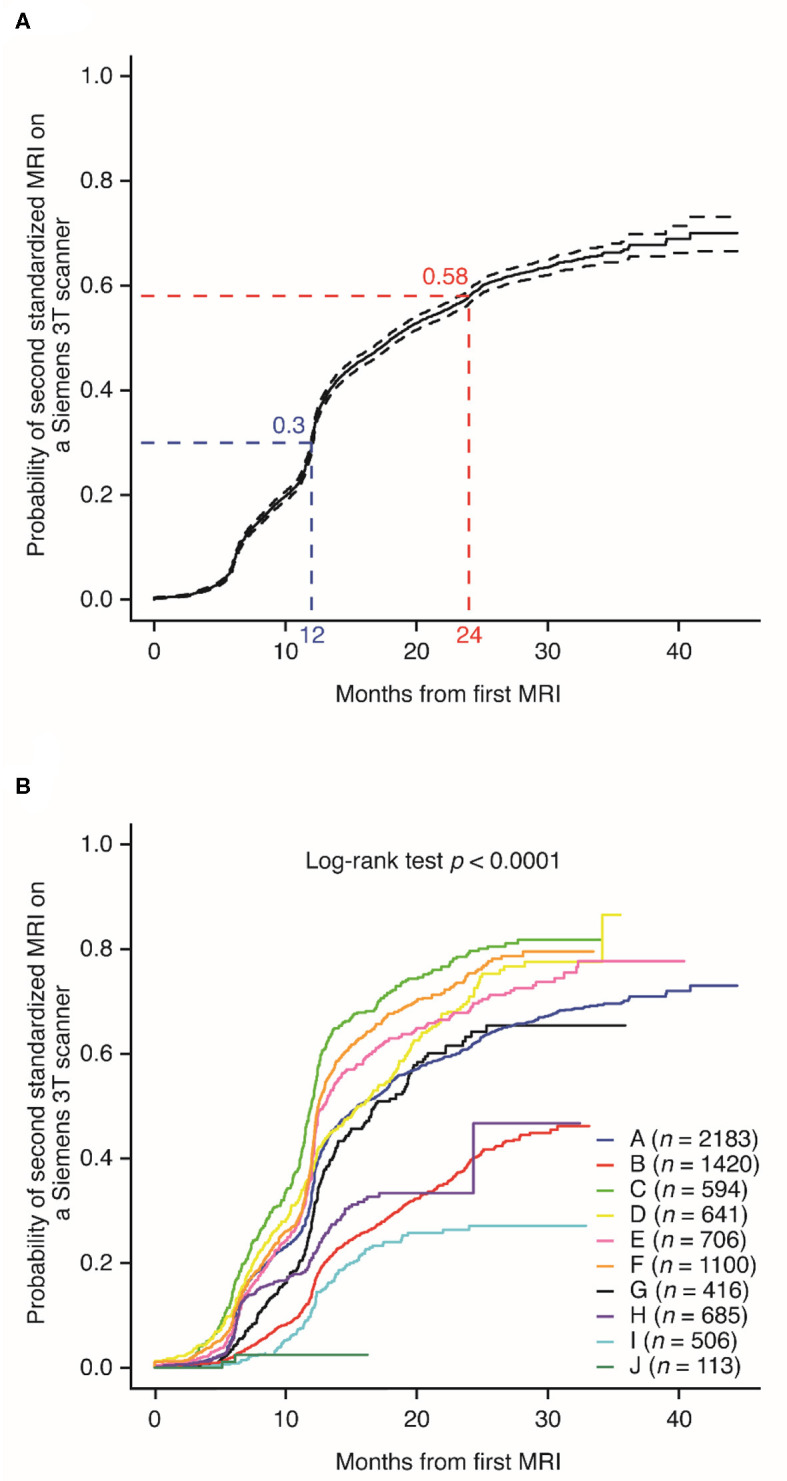
Probability of completing a follow-up standardized magnetic resonance imaging on a Siemens 3T scanner, **(A)** overall and **(B)** by individual Multiple Sclerosis Partners Advancing Technology and Health Solutions center.

After completing their initial standardized 3T brain MRI, 30% and 58% of patients in MS PATHS completed a follow-up standardized 3T brain MRI within 12 and 24 months, respectively (Figure 2A). The median time between the two standardized 3T brain MRIs was ~18 months. The median time to complete a follow-up standardized 3T brain MRI varied considerably among the participating healthcare institutions, ranging from 12 to 24 months (Figure 2B; *n* = 721; *p* < 0.0001). In four institutions, half of the patients had not yet had a second standardized 3T brain MRI.

DMT use varied across the participating sites, in terms of both whether or not a patient was treated with a DMT (χ^2^ = 163.3; *p* < 0.0001) and which DMT was prescribed (χ^2^ = 2,584.6; *p* < 0.0001; Table 8).

**Table 8:** Distribution of DMT use by participating healthcare institutions at initial assessment^*^.

		**Institution**									
**DMT, *n* (%)**	**Overall (*n* = 14,643)**	**A (*n* = 1,581)**	**B (*n* = 1,061)**	**C (*n* = 572)**	**D (*n* = 1,722)**	**E (*n* = 3,690)**	**F (*n* = 777)**	**G (*n* = 1,833)**	**H (*n* = 1,607)**	**I (*n* = 621)**	**J (*n* = 1,179)**
Alemtuzumab	230 (1.6)	12 (0.1)	3 (0.0)	4 (0.0)	103 (0.7)	19 (0.1)	14 (0.1)	4 (0.0)	28 (0.2)	0 (0.0)	43 (0.3)
Azathioprine	6 (0.0)	0 (0.0)	0 (0.0)	0 (0.0)	0 (0.0)	0 (0.0)	0 (0.0)	0 (0.0)	2 (0.0)	2 (0.0)	2 (0.0)
Cyclophosphamide	2 (0.0)	0 (0.0)	1 (0.0)	0 (0.0)	0 (0.0)	1 (0.0)	0 (0.0)	0 (0.0)	0 (0.0)	0 (0.0)	0 (0.0)
Daclizumab	37 (0.3)	1 (0.0)	0 (0.0)	0 (0.0)	0 (0.0)	2 (0.0)	0 (0.0)	4 (0.0)	0 (0.0)	6 (0.0)	24 (0.2)
Dimethyl fumarate	1,966 (13.4)	152 (1.0)	159 (1.1)	67 (0.5)	179 (1.2)	641 (4.4)	104 (0.7)	317 (2.2)	115 (0.8)	104 (0.7)	128 (0.9)
Fingolimod	1,645 (11.2)	148 (1.0)	138 (0.9)	70 (0.5)	241 (1.6)	484 (3.3)	74 (0.5)	62 (0.4)	101 (0.7)	105 (0.7)	222 (1.5)
Glatiramer acetate	1,760 (12)	324 (2.2)	63 (0.4)	88 (0.6)	159 (1.1)	395 (2.7)	95 (0.6)	330 (2.3)	158 (1.1)	39 (0.3)	109 (0.7)
Immunoglobulin G	12 (0.1)	0 (0.0)	3 (0.0)	0 (0.0)	2 (0.0)	6 (0.0)	1 (0.0)	0 (0.0)	0 (0.0)	0 (0.0)	0 (0.0)
Interferon beta-1a	1,088 (7.4)	171 (1.2)	50 (0.3)	44 (0.3)	67 (0.5)	295 (2.0)	41 (0.3)	129 (0.9)	225 (1.5)	31 (0.2)	35 (0.2)
Interferon beta-1b	245 (1.7)	12 (0.1)	10 (0.1)	11 (0.1)	13 (0.1)	13 (0.1)	9 (0.1)	14 (0.1)	130 (0.9)	16 (0.1)	17 (0.1)
Interferon beta-other	16 (0.1)	2 (0.0)	4 (0.0)	0 (0.0)	2 (0.0)	6 (0.0)	0 (0.0)	2 (0.0)	0 (0.0)	0 (0.0)	0 (0.0)
Methotrexate	27 (0.2)	1 (0.0)	2 (0.0)	0 (0.0)	0 (0.0)	21 (0.1)	0 (0.0)	2 (0.0)	0 (0.0)	1 (0.0)	0 (0.0)
Mitoxantrone	6 (0.0)	0 (0.0)	1 (0.0)	0 (0.0)	2 (0.0)	1 (0.0)	0 (0.0)	0 (0.0)	0 (0.0)	1 (0.0)	1 (0.0)
Mycophenolate mofetil	24 (0.2)	3 (0.0)	1 (0.0)	0 (0.0)	2 (0.0)	13 (0.1)	0 (0.0)	5 (0.0)	0 (0.0)	0 (0.0)	0 (0.0)
Natalizumab	1,384 (9.5)	139 (0.9)	155 (1.1)	53 (0.4)	271 (1.9)	263 (1.8)	65 (0.4)	217 (1.5)	95 (0.6)	34 (0.2)	92 (0.6)
Ocrelizumab	748 (5.1)	27 (0.2)	60 (0.4)	64 (0.4)	140 (1.0)	173 (1.2)	21 (0.1)	108 (0.7)	23 (0.2)	27 (0.2)	105 (0.7)
Ofatumumab	1 (0.0)	0 (0.0)	0 (0.0)	0 (0.0)	1 (0.0)	0 (0.0)	0 (0.0)	0 (0.0)	0 (0.0)	0 (0.0)	0 (0.0)
Peginterferon beta-1a	159 (1.1)	14 (0.1)	15 (0.1)	17 (0.1)	5 (0.0)	26 (0.2)	5 (0.0)	38 (0.3)	22 (0.2)	7 (0.0)	10 (0.1)
Rituximab	272 (1.9)	45 (0.3)	67 (0.5)	18 (0.1)	18 (0.1)	15 (0.1)	7 (0.0)	65 (0.4)	31 (0.2)	3 (0.0)	3 (0.0)
Teriflunomide	562 (3.8)	54 (0.4)	39 (0.3)	41 (0.3)	85 (0.6)	76 (0.5)	39 (0.3)	42 (0.3)	104 (0.7)	28 (0.2)	54 (0.4)
Other	1,077 (7.4)	139 (0.9)	74 (0.5)	20 (0.1)	110 (0.8)	298 (2.0)	68 (0.5)	98 (0.7)	103 (0.7)	58 (0.4)	109 (0.7)
None	3,341 (22.8)	337 (2.3)	214 (1.5)	75 (0.5)	317 (2.2)	922 (6.3)	234 (1.6)	395 (2.7)	465 (3.2)	159 (1.1)	223 (1.5)
Missing	35 (0.2)	0 (0.0)	2 (0.0)	0 (0.0)	5 (0.0)	20 (0.1)	0 (0.0)	1 (0.0)	5 (0.0)	0 (0.0)	2 (0.0)

*DMT, disease-modifying therapy*.

* *Likelihood ratio (DMT × site) χ^2^ = 2,584.6; p < 0.0001*.

### Heterogeneity Relative to Phase 3 MS RCTs

The patients in MS PATHS were significantly older and had a longer disease duration than the patients from the pooled RCTs (Table 9). In addition, the proportion of patients with no relapses in the last 12 months was higher in MS PATHS (54.4%) than in the pooled RCTs (1.4%). The logistic regression models predicting membership in MS PATHS resulted in a C statistic of 0.91 vs. the pooled RCTs. A C statistic of 1.0 indicates no overlap; 0.91 indicates that RCT patients were substantially distinct from patients in MS PATHS. Propensity score 1:1 matching of the 8,708 patients with RRMS in MS PATHS and 6,574 patients with RRMS in the pooled RCTs yielded 1,922 patients from each sample that were able to be matched (Table 9).

**Table 9:** Demographic and clinical characteristics of patients with RRMS in MS PATHS and Biogen phase 3 RCTs (25–29).

	**Unmatched population**			**Propensity score-matched population**		
Variable^*^	**MS PATHS**	**RCTs**	**Cohen's**	**MS PATHS**	**RCTs**	**Cohen's**
			***d* value**			***d* value**
*N*	8,708	6,574		1,922	1,922	
Age (years)	45.0 (12.2)	37.4 (8.9)	−0.71	40.7 (11.3)	40.3 (8.7)	−0.04
Male (%)	23.9	28.3	0.10	25.4	26.4	0.02
Body mass index (kg/m^2^)	28.9 (7.0)	25.1 (5.4)	−0.60	27.9 (6.3)	27.8 (6.8)	−0.01
MS duration (years)	11.3 (9.0)	4.8 (5.0)	−0.90	7.2 (6.3)	7.1 (6.3)	−0.01
Manual dexterity test/9-Hole Peg Test (seconds)^†^	25.9 (5.9)	22.1 (6.1)	−0.63	25.1 (4.7)	25.3 (8.0)	0.03
25-foot walking speed test/Timed 25-Foot Walk (seconds)^†^	6.6 (3.2)	6.4 (4.5)	−0.04	6.5 (3.0)	6.5 (3.3)	0.01
Number of relapses in the past 12 months						
NA	0.8	4.6	0.24	1.9	2	0.01
0	54.4	1.4	−1.46	4.8	4.8	0
1	23.8	58.8	0.76	54.9	54.1	−0.02
2	12.6	29	0.41	26.5	26.7	0
≥3	8.4	6.1	−0.09	11.8	12.4	0.02

*MS, multiple sclerosis; MS PATHS, Multiple Sclerosis Partners Advancing Technology and Health Solutions; NA, not applicable; RCT, randomized clinical trial; RRMS, relapsing-remitting multiple sclerosis*.

* *Data are reported as mean (SD) or n (%) for continuous and categorical variables, respectively. Cohen's d values represent standardized mean or proportion differences (i.e., effect sizes). An absolute value of % d >10 is considered as clinically meaningful*.

† *The manual dexterity test and 25-foot walking speed test were administered in MS PATHS and the 9-Hole Peg Test and Timed 25-Foot Walk were administered in the RCTs*.

### Research Utilization of LHS Data

The governing subcommittee has approved 58 data use requests and one sample use request. Based on these requests, 78 conference abstracts and manuscripts have been published. Two National Institutes of Health grants have been funded.

## Discussion

The Institute of Medicine, a member of the US National Academies, recommended foundational elements for an LHS (10) as an approach to address recognized challenges, including the need for consistent quality and efficiency, in the US healthcare system. These challenges are also recognized by healthcare systems and regulatory bodies outside of the United States such as the European Medicines Agency (2, 4, 33–35). As the first LHS in MS, MS PATHS was designed to incorporate each of the foundational elements outlined in the introduction.

There were three initial observations from MS PATHS. The first relates to the characteristics of patients with MS. Although the patients in MS PATHS demonstrate demographic and disease characteristics typical of other large MS cohorts, the clinical characteristics from patients with RRMS in MS PATHS are partially non-overlapping with the patients enrolled in clinical trials (25–29), thus reflecting a broader population. The patients in MS PATHS exhibited a broader range of ages, disability levels, disease duration, and DMT use, presumably because MS PATHS aims to enroll all patients with a diagnosis of MS, including clinically isolated syndrome, with no additional inclusion or exclusion criteria (e.g., requiring a relapse in the past 12 months, as is typically required in pivotal RCTs). MS PATHS is more racially diverse than previously reported trials; white patients make up 79.5% of the US MS PATHS population in comparison with 93.7% of the oral DMT clinical trial study population (36). The minimal inclusion criteria and the robust enrollment rates suggest that studies using the MS PATHS population will generate data that will be more generalizable than data from clinical trials, in which enrollment is more selective. It is also likely that patient characteristics obtained from MS PATHS will be more representative of the broader MS population than data from registries where a smaller subset of patients from a clinical practice in one location is selected for inclusion in the cohort (37).

The second observation is the demonstrated feasibility of real-time quantitative patient phenotyping as part of clinical practice. As of August 5, 2019, 88.4% of patients who gave permission for their clinical data to be used for research have completed at least one self-administered neuroperformance assessment. Also, standardized MRI acquisition sequences were incorporated into clinical brain MRI protocols, with 98% of scans passing the quality control assessments.

The third observation relates to the variability in practice patterns observed across participating healthcare institutions. We observed a significant inter-institution variability in the rate of return for follow-up and the average interval between assessments captured in MS PATHS, indicating that there is no uniform standard for visit frequency in the network. Follow-up interval is important when determining outcomes because visit frequency may influence observed event frequency, depending on how the information is captured. For example, individuals with MS may not always present to the physician during a relapse (38).

MS PATHS has experienced challenges and learnings during the initial implementation, including:

(1) Creating trust in an academic–industry collaboration: The initial collaborators were excited about the vision of MS PATHS; however, there were questions on how to build trust in a multi-party collaboration with a biopharmaceutical company. The initial collaborators recognized that a transparent governance process would be key to engender trust among the participants and in the scientific community at large. All parties quickly aligned on a vision of using the LHS concept to generate evidence to improve outcomes for patients with MS as well as the guiding principles (Table 1). The governance model was then developed over the course of six all-day meetings to enable all parties to fulfill the shared vision and operate by the guiding principles.

(2) Aligning on which data to collect and how to standardize data collection: Given the broad research goals of MS PATHS, the potential data to collect were vast. In the initial planning discussions, it became clear that standardizing and collecting all potential data would not fit into the clinical workflow. Data were prioritized in terms of time and utility. The goal was to keep MSPT administration no longer than 30 minutes for the average patient with MS, keep the brain MRI total scan time unchanged, and add no additional time to a clinician's clinical documentation. Any suggested patient-reported question or scale was weighed in terms of time to complete and its potential utility for clinical care and research. Implementing axial reconstruction of the standardized three-dimensional sequences allowed radiologists/neurologists to view scans as they normally would without the need for duplicative MRI acquisitions. Also, collaborating with Siemens Healthineers allowed the sequences to be optimized based on feedback from MS PATHS radiologists to enable broad adoption in the network.

(3) Implementation of common health IT platforms: Generating data in real time for clinical decision-making and providing pseudonymized data for research involved major technical challenges. We started with a basic design of a health information exchange platform and iterated after receiving feedback from key IT stakeholders at the founding institutions. Because IT architecture is different in each medical center, interfaces for the IT platforms need to be customized for each medical center to ensure compatibility and adherence to security requirements. The current implementation maintains consistent data handling and processing across the network but flexes to allow for variability in the interfaces and whether a push or pull model is optimal for each individual medical center. This type of model was aided by the increasing adoption of common healthcare data standards across healthcare in general.

(4) Ensuring compliance with US and EU data privacy regulations: MS PATHS was designed to be both HIPAA and GDPR compliant. In MS PATHS, patient data are only shared for research purposes, with patient consent. Trusted third parties serve as intermediaries to remove patient identifiers before the data are aggregated for research under appropriate contractual arrangements. Garnering acceptance and approval from a myriad of stakeholders at each institution is equally as important as a sound program design. MS PATHS has shown that with a sound program design, stakeholder engagement, and patient permission, data aggregation is feasible to conduct under both HIPAA and GDPR.

(5) Enabling efficient data sharing: Our chosen hub-and-spoke data sharing model simplified contractual relationships in MS PATHS. Each healthcare institution negotiated directly with Biogen rather than having to enter into a multi-lateral negotiation for the initial contract and any subsequent amendments. All stakeholders acknowledged the logistical benefits of this model. However, it required key clauses on the scope of data sharing, data permissions, and intellectual property to be uniform across all contracts and informed consent forms and for all parties to be comfortable that we could not accommodate one-off deviations if we were to maintain an effective hub-and-spoke model.

(6) Re-engineering of clinical workflows involved all aspects of clinic operations: One example of how MS clinics needed to change their operations was the incorporation of the MSPT into the clinical workflow. This necessitated designating space in the clinic as a MSPT testing area, training staff on the MSPT, introducing MS patients to a new aspect of their visit, and adjusting patient arrival times whether formally through a new appointment time or reminders to arrive 30 min before their scheduled visit with their healthcare provider. The workflow challenges differed somewhat for implementing the MSPT, implementing the standardized MRI protocol, and research workflows such as biobanking. Enthusiasm for the LHS tenets and the project sustained commitment from academic and industry project leaders, overcoming a myriad of challenges. The network investigators also committed to sharing best practices through conference calls, investigator meetings, and site visits. For example, an early MSPT implementation insight shared across the network was that patients generally have a better testing experience if they take the test without staff, a family member, or a caregiver by their side.

(7) Real-life experience highlighted gaps in original technical assumptions: Some design issues that have emerged and are being corrected include issues with MSPT functionality (e.g., lower-than-expected contrast sensitivity test completion rates) and issues with optimizing data collection methods for certain variables (e.g., comorbidities, medication start/stop dates). Among the advantages of an academic and industry partnership model are the real-time and continuous feedback, re-assessment, and refinement of data gathering and overall strategy that facilitate continuous improvement. One example of this feedback loop is the creation of a MS SmartForm by one of the MS PATHS institutions that is freely available in EPIC foundation to all EPIC users. This SmartForm will facilitate the standardization of key MS-related variables such as relapses and disease-modifying therapy start/stop dates to primarily aid clinical care, but with the secondary benefit of improving data quality for research. The US MS PATHS centers are currently in various stages of SmartForm implementation, while we are coordinating with the EU MS PATHS centers to harmonize as much as possible.

(8) Incorporating the voice of the patient: Patients have always been identified as a key stakeholder in MS PATHS. During the design phase, the investigators engaged a local patient advisory group, when available, on the MS PATHS concept. The MSPT was designed to facilitate the voice of the patient being incorporated into the clinical visit through the neuroperformance modules and 12 domains of quality-of-life as assessed by the Neuro-QoL. Prior to the initial deployment of the MSPT in MS PATHS, qualitative feedback was obtained by the patients in an initial usability study (11). However, there is still more to be done to engage patients and complete the feedback loop as envisioned in a LHS (10). To start, the network is working to generate regular patient newsletters that provide updates on MS PATHS and summarize recent research presented at conferences or published in journals. Each MS center will provide these updates *via* their routine patient communication channels. We are also working on an updated publicly facing website that would also have this information.

(9) Moving from clinical implementation to clinical decision-making: Our efforts to date have been focused on the initial implementation and data collection as highlighted in this paper. The network is now shifting focus to evidence generation that will enable the data collected in MS PATHS to be used more routinely for clinical decision-making. Key next topics include understanding clinical cutoffs for the MSPT and MRI metrics, looking at practice heterogeneity across the network to identify best practices, and exploring feedback loops for investigators to more easily use the data for quality improvement.

Despite the promise of this research, there are limitations to be considered. Data missingness may be non-random. For example, patients with more disability may be less likely to have an MRI or complete the MSPT. The participating centers are referral centers and may not fully represent broader MS populations. For example, the rates of patients with progressive disease on a DMT may be higher than expected, and incorporation of the SmartForm data may provide additional insight. Still the MS PATHS population represents an improvement over the populations included in clinical trials as it is more racially and clinically diverse.

Another limitation is related to the restriction of standardized MRI studies to Siemens 3T scanners. This was intentional in order to remove scanner and manufacturer variability from the derived metrics as the initial step in delivering point-of-care MRI-based metrics to the clinician. As such, the time to follow-up MRI does not take into account the MRI exams acquired within each institution on non-Siemens 3T MRI scanners or using different (non-standardized) acquisition sequences or an MRI obtained outside the healthcare institution. Therefore, differences in MRI follow-up time between centers may reflect variations in the use of Siemens 3T scanners and variation in the percentage of patients who obtain MRI exams outside of the healthcare institution. A few participating centers image their patients with MS exclusively on Siemens 3T scanners, while others use a wide variety of scanner types across internal and external imaging facilities. Translation of the imaging methods to non-Siemens 3T scanners is planned.

The initial implementation of MS PATHS has been successful overall, although we are continuously optimizing aspects of our operations. We anticipate its value to grow with time as large-scale, longitudinal, and multidimensional quantitative data accumulate. These data will potentially drive new insights and aid in the development and the validation of new technologies. In particular, incorporating point-of-care quantitative MRI-based metrics in clinical practice has the potential to transform precision medicine to the same degree that MRI-based metrics have advanced clinical trials.

Another important direction for future work in MS PATHS is defining individual patient outcomes using quantitative standardized data. As MS PATHS data accumulate, it may be possible to define complete MS disease control or actionable change based on quantitative monitoring. Further, in the future, decision support based on advanced analytic methods could help neurologists care for individuals with MS in the context of a large number of DMTs and other therapeutic choices. Data accumulated in MS PATHS could support the development of predictive models and decision support systems.

The LHS concept, exemplified by MS PATHS, could accelerate translational research in MS as evidenced by the number of active proposals and grant approvals. Tens of thousands of patients may be required to unravel the biological basis for MS heterogeneity and develop personalized medicine based on individual patient characteristics. Using quantitative data derived from clinical, imaging, and laboratory assessments generated during patient care could drastically reduce the cost of, and thus make feasible, such translational research studies.

## Data Availability Statement

The datasets presented in this article are not readily available because MS PATHS is an ongoing study. Requests to access the datasets should be directed to datasharing@biogen.com.

## Ethics Statement

The studies involving human participants were reviewed and approved by Ethics committees or institutional review boards at the following participating centers: Cleveland Clinic, Cleveland, OH, USA; Johns Hopkins University, Baltimore, MD, USA; New York University, New York, NY, USA; OhioHealth, Columbus, OH, USA; Washington University in St. Louis, St. Louis, MO, USA; University of Rochester Medical Center, Rochester, NY, USA; Cleveland Clinic Lou Ruvo Center for Brain Health, Las Vegas, NV, USA; University Hospital of Giessen and Marburg, Marburg, Germany; Vall d'Hebron University Hospital, Barcelona, Spain; and University Hospital Carl Gustav Carus, Dresden, German. The patients/participants provided their written informed consent to participate in this study.

## Author Contributions

JW, CM, EF, and RR conceived and designed the data plan. CM and FP conducted the analyses. EM, RB, JW, and RR led the writing of the manuscript. RR had final responsibility for the decision to submit for publication. All authors provided critical feedback and helped shape the research, analysis, manuscript, had full access to the study data for interpretation and drafting of the report, and contributed to the interpretation of the findings. Editorial support was provided to authors and funded by the study funder. All decisions relating to manuscript writing and content were made jointly by the authors.

## Conflict of Interest

The participating healthcare institution of each investigator receives financial compensation for data shared and biosamples collected as part of this program based on fair market value. EM reports research support from Biogen (MS PATHS and investigator-initiated studies) and Sanofi-Genzyme for investigator-initiated studies, serving as site principal investigator for a Sun Pharma study, free medication from Teva Neuroscience for use in a clinical trial, for which she is principal investigator, and royalties for editorial duties from UpToDate. RB reports consulting fees from Biogen, Genentech/Roche, Novartis, and Sanofi-Genzyme, research support from Biogen, and personal and institutional equity ownership related to the MSPT licensed to Biogen and Qr8 by Cleveland Clinic. JW, CM, EF, BK, FP, and RR are employees of and hold stock/stock options in Biogen. TB reports research support from Avid/Lilly and Roche, travel support from Biogen, and serving as clinical trial investigator for Avid/Lilly, Biogen, and Roche. CH reports speaking and consulting fees from Biogen, EMD Serono, Genentech, Genzyme, and Novartis, research support from Biogen, Genentech, and Patient-Centered Outcomes Research Institute, and research support to her institution, Cleveland Clinic, from Biogen. MH reports research support from Biogen, Chugai, National Institutes of Health (NeuroNEXT), Novartis, and Patient-Centered Outcomes Research Institute. II reports funding from Biogen in the form of a contract with her employer, Johns Hopkins University (JHU), to support effort on this project, serving as principal investigator on a research grant from Siemens paid to JHU for an unrelated project, and consultant fees from Alexion. SJ reports speaker fees from St. Jude Hospital, travel support from Siemens, grant support from Biogen for MS PATHS, and grant support from St. Jude Hospital. HK reports research support from Novartis and traveling and speaking/consulting fees and/or honoraria from Novartis, Biogen, Teva, IXICO, and Siemens. LK reports consulting fees from Biogen, Gerson Lehrman, Novartis, and Sanofi, grant support from Biogen, and receipt of royalties for the Fatigue Severity Scale licensed to various biopharmaceutical entities. YL declares no competing interests. XM reports speaker fees/travel expenses for scientific meetings or steering committees/advisory boards for clinical trials for Actelion, Biogen, Celgene, EXCEMED, Genzyme, Merck Serono, MS International Foundation, National MS Society, Novartis, Sanofi-Genzyme, Roche, and Teva. RN reports consulting fees and/or honoraria from Alexion, Alkermes, Biogen, Celgene, EMD Serono, Genentech, Genzyme, NervGen, Novartis, TG Therapeutics, and Third Rock Ventures. JN reports consulting/speaking honoraria from Biogen, EMD Serono, Genentech, Genzyme, and Novartis. AR serves on scientific advisory boards for Bayer, Biogen, Icometrix, Olea Medical, and SyntheticMR, is a consultant/speaker for Novartis, Roche, and Sanofi-Genzyme, and receives research support from Biogen. MS reports research funding for the conduct of MS PATHS from Biogen. BT reports advisory boards for Bayer, Biogen, CSL Behring, Genzyme, Grifols, Novartis, and UCB, speaker honoraria from Bayer, Biogen, CSL Behring, Genzyme, Grifols, Novartis, and Octapharma, consulting fees from Bayer, Biogen, CSL Behring, Genzyme, Grifols, Novartis, and UCB, and research support from Biogen and Novartis. MTin reports consulting/speaking honoraria from Almirall, Bayer Schering, Biogen, Genzyme, Merck Serono, Novartis, Roche, Sanofi, and Teva, grants from Biogen, Genzyme, and Novartis, and serving as co-editor of Multiple Sclerosis Journal – Experimental, Translational, and Clinical. MTiv reports funding from Biogen in the form of a contract with her employer, University of Rochester, to support efforts on MS PATHS. TZ reports speaker fees/travel expenses for scientific meetings or steering committees/advisory boards for clinical trials for Almirall, Bayer, Biogen, Celgene, Genzyme, Gilead, Merck Serono, Novartis, Roche, and Teva and grant support from BAT, Biogen, Genzyme, Merck Serono, Novartis, and Roche. The handling editor declared a past co-authorship with AR.

## Abbreviations

DMT: disease-modifying therapy
EMR: electronic medical record
FLAIR: fluid-attenuated inversion recovery
IQR: interquartile range
IT: information technology
LHS: learning health system
MPRAGE: magnetization-prepared rapid gradient-echo imaging
MRI: magnetic resonance imaging
MS: multiple sclerosis
MS PATHS: Multiple Sclerosis Partners Advancing Technology and Health Solutions
MSPT: Multiple Sclerosis Performance Test
NA: not applicable
Neuro-QoL: Quality of Life in Neurological Disorders
PPMS: primary progressive multiple sclerosis
PRMS: progressive relapsing multiple sclerosis
RCT: randomized controlled clinical trial
RRMS: relapsing-remitting multiple sclerosis
SPMS: secondary progressive multiple sclerosis.
