# Supplementary material for: Harnessing Real-World Data to Inform Decision-Making: Multiple Sclerosis Partners Advancing Technology and Health Solutions (MS PATHS)
Source: Front Neurol. 2020 Aug 7;11:632. doi: 10.3389/fneur.2020.00632 (PMC7426489; doi:10.3389/fneur.2020.00632)
Supplement: Supplementary file 1 [file Data_Sheet_1.PDF]

Overview

The MRI initiative of MS PATHS generates quantitative metrics of MS disease activity and progression. Initial development work is focused on new T2 lesion counts and brain atrophy. It is our goal to have the calculations integrated into the radiology workflow, and the results available to the radiologist and the neurologist at the point of care. MS PATHS also provides researchers across participating centers with a centralized database of standardized, comparable MRI data. To achieve these goals, participating centers in MS PATHS have agreed to standardized MPRAGE and 3D FLAIR sequences that are collected as part of each institution's routine MS care imaging exams. In the initial stage of MS PATHS, the quantitative MRI metrics will be under development. We aim to implement a prototype system at select MRI testing centers in the MS PATHS network later in the course of the project. Participating centers in MS PATHS have agreed to also use specific keys in their radiology reporting macros to enable capture of lesion count data from the radiology reports.

Protocol

The MS PATHS sequences should be included in each MRI protocol used for routine clinical MS monitoring. Both the MPRAGE and the 3D FLAIR scans for MS PATHS are optimized versions of Siemens product sequences (see *Appendix A*). Acquisition time for the MPRAGE is 5:12 min:s, and the 3D FLAIR is 6:27 min:s. The voxel resolution for both scans is 1x1x1 mm^3. Ideally, the MPRAGE and 3D FLAIR should be the first two sequences in the study. The MPRAGE must be acquired pre-contrast; the 3D FLAIR may be acquired post-contrast. The field of view should be set up to ensure full brain coverage. It is recommended to use the AutoAlign scout included with the MS PATHS sequences (if not already routinely used at your healthcare institution). See *Appendix B* for protocol set-up tips and common issues.

To facilitate the identification of these scans in the hospital radiology systems, the sequences will be installed with specific names, i.e., "3D-T1-MS-P" and "3D-FLAIR-MS-P". **These names should never be changed**. It is equally important that the sequence parameters for the MRPAGE and 3D FLAIR are not altered, and that the imaging procedures remain as consistent as possible over time for the patient. Ideally, MS PATHS participants should be imaged on the same scanner using the same head coil at each visit.

Each healthcare institution should follow all other aspects of their regular routine for scanning patients, for example, ensuring patient comfort, positioning the patient, etc. Image quality checks and re-scanning in the event of excessive patient motion should be performed in the same manner as usually done for routine MS imaging.

QA/QC

As part of the full MS PATHS implementation, the IT infrastructure enables pseudoanyonmized MS PATHS images to be pushed from the clinic into a cloud-based data repository called the Learning Health System (LHS). During the set-up phase, each healthcare institution should send a pseudoanyonmized MS PATHS image set (i.e., the 3D FLAIR and MPRAGE) every week to Biogen for a quality analysis. This sample image set can be selected

randomly or based on concerns about image quality. In either case, it should be chosen from an MS PATHS patient scanned within that particular week. The scans will be sent to Biogen for review via site-specific instructions to be provided by email. After the initial set-up phase, Biogen will continue to monitor and review a subset of incoming images. The images will be reviewed in order to:

- track scanner changes that may affect the MS PATHS sequences (such as software upgrades or the use of a different scanner to collect the MS PATHS sequences)
- ensure there are no changes to the sequence/protocol parameters or names
- ensure complete brain coverage
- evaluate image quality (motion, aliasing, etc)

Artifacts

Motion in 3D acquisitions is quite different than motion in 2D acquisitions. An example of motion is illustrated in *Figure 1, Appendix C*. We will also be reviewing 3D FLAIR images for insufficient nulling of the CSF (*Figure 2, Appendix C*) and banding artifacts (*Figure 3, Appendix C).*

Notification of poor image quality

During the set-up phase, you will receive a response (*example attached in Appendix D*) within one week if the QA/QC scan did not pass the quality review. If needed, our MS PATHS imaging team will work with you to help resolve any issues. After the initial set-up phase, a member of the MS PATHS imaging team will contact your site's MS PATHS Lead Radiologist if any systematic issues are noted during ongoing image QA/QC.

Support

The MS PATHS imaging team is available for questions regarding the protocol. Please contact us by email if you have any questions or if you are aware of any upcoming changes to your system, which may affect the consistency of MS PATHS images over time.

Any non-MS PATHS related issues (general scanner issues, coil problems, etc) should be handled by the existing Siemens service infrastructure. Siemens will provide the usual upgrade support for user protocol conversion during training by an applications specialist, and Biogen will provide additional upgrade support of MS PATHS protocols.

Appendix A – 3D-T1 and 3D-FLAIR Protocols

Following 7 pages. Example from software version VE11.

SIEMENS MAGNETOM Skyra

Table of contents

\\USER
CCF_MS_Brain_MS-PATHS
CCF_Dot_MS_Brain_Project
protocol
AAHScout *
3D T1 - MS-P *
3D FLAIR - MS-P *

SIEMENS MAGNETOM Skyra

\\USER\CCF_MS_Brain_MS-PATHS\CCF_Dot_MS_Brain_Project\protocol\AAHScout *

TA: 0:14 PM: ISO Voxel size: 1.6×1.6×1.6 mmPAT: 3 Rel. SNR: 1.00 : fl

Properties

Prio recon	Off
Load images to viewer	On
Inline movie	Off
Auto store images	On
Load images to stamp segments	Off
Load images to graphic segments	Off
Auto open inline display	Off
Auto close inline display	Off
Start measurement without further preparation	On
Wait for user to start	Off
Start measurements	Single measurement

Routine

Slab group	1
Slabs	1
Dist. factor	20 %
Position	L0.0 P20.0 H0.0 mm
Orientation	Sagittal
Phase enc. dir.	A >> P
Phase oversampling	0 %
Slice oversampling	0.0 %
Slices per slab	128
FoV read	260 mm
FoV phase	100.0 %
Slice thickness	1.6 mm
TR	3.15 ms
TE	1.37 ms
Averages	1
Concatenations	1
Filter	Distortion Corr.(2D), Prescan Normalize
Coil elements	HE1-4

Contrast - Common

TR	3.15 ms
TE	1.37 ms
Flip angle	8 deg

Contrast - Dynamic

Averages	1
Averaging mode	Short term
Reconstruction	Magnitude
Measurements	1

Resolution - Common

FoV read	260 mm
FoV phase	100.0 %
Slice thickness	1.6 mm
Base resolution	160
Phase resolution	100 %
Slice resolution	69 %
Phase partial Fourier	6/8
Slice partial Fourier	6/8
Trajectory	Cartesian

Resolution - iPAT

PAT mode	GRAPPA
Accel. factor PE	3
Ref. lines PE	24
Accel. factor 3D	1
Reference scan mode	Integrated

Resolution - Filter Image

Image Filter	Off
Distortion Corr.	On
Mode	2D
Unfiltered images	Off
Prescan Normalize	On
Unfiltered images	Off
Normalize	Off
B1 filter	Off

Resolution - Filter Rawdata

Raw filter	Off
Elliptical filter	Off

Geometry - Common

Slab group	1
Slabs	1
Dist. factor	20 %
Position	L0.0 P20.0 H0.0 mm
Orientation	Sagittal
Phase enc. dir.	A >> P
Slice oversampling	0.0 %
Slices per slab	128
FoV read	260 mm
FoV phase	100.0 %
Slice thickness	1.6 mm
TR	3.15 ms
Multi-slice mode	Sequential
Series	Ascending
Concatenations	1

Geometry - AutoAlign

Slab group	1
Position	L0.0 P20.0 H0.0 mm
Orientation	Sagittal
Phase enc. dir.	A >> P
Initial Position	Isocenter
L	0.0 mm
P	0.0 mm
H	0.0 mm
Initial Rotation	0.00 deg
Initial Orientation	Transversal

System - Miscellaneous

Positioning mode	ISO
Table position	H
Table position	0 mm
MSMA	S - C - T
Sagittal	R >> L
Coronal	A >> P
Transversal	F >> H
Coil Combine Mode	Adaptive Combine
Save uncombined	Off
Matrix Optimization	Off
Coil Select Mode	On - AutoCoilSelect

- 2 -

SIEMENS MAGNETOM Skyra

System - Adjustments

B0 Shim mode	Tune up
B1 Shim mode	TrueForm
Adjust with body coil	Off
Confirm freq. adjustment	Off
Assume Dominant Fat	Off
Assume Silicone	Off
Adjustment Tolerance	Auto

System - Adjust Volume

Position	Isocenter
Orientation	Transversal
Rotation	0.00 deg
A >> P	263 mm
R >> L	350 mm
F >> H	350 mm
Reset	Off

System - Tx/Rx

Frequency 1H	123.138297 MHz
Correction factor	1
Gain	Low
Img. Scale Cor.	1.000
Reset	Off
? Ref. amplitude 1H	0.000 V

Physio - PACE

Resp. control	Off
Concatenations	1

Inline - Common

Flip angle	8 deg
Measurements	1
Time to center	6.2 s

Inline - Inline

Subtract	Off
Measurements	1
StdDev	Off
Save original images	On

Inline - MIP

MIP-Sag	Off
MIP-Cor	Off
MIP-Tra	Off
MIP-Time	Off
Save original images	On

Inline - Composing

Distortion Corr.	On
Mode	2D
Unfiltered images	Off

Sequence - Part 1

Introduction	On
Dimension	3D
Asymmetric echo	Weak
Contrasts	1
Multi-slice mode	Sequential
Bandwidth	540 Hz/Px

Sequence - Part 2

RF pulse type	Fast
Gradient mode	Normal

Sequence - Part 2

Excitation	Non-sel.
RF spoiling	On

Sequence - Assistant

Mode	Min flip angle
Min flip angle	90.0 deg

SIEMENS MAGNETOM Skyra

\\USER\CCF_MS_Brain_MS-PATHS\CCF_Dot_MS_Brain_Project\protocol\3D T1 - MS-P *

TA: 5:12 PM: ISO Voxel size: 1.0×1.0×1.0 mmPAT: 2 Rel. SNR: 1.00 : tfl

Properties

Prio recon	Off
Load images to viewer	On
Inline movie	Off
Auto store images	On
Load images to stamp segments	On
Load images to graphic segments	On
Auto open inline display	Off
Auto close inline display	Off
Start measurement without further preparation	Off
Wait for user to start	Off
Start measurements	Single measurement

Routine

Slab group	1
Slabs	1
Dist. factor	50 %
Position	Isocenter
Orientation	Sagittal
Phase enc. dir.	A >> P
AutoAlign	Head > Basis
Phase oversampling	0 %
Slice oversampling	0.0 %
Slices per slab	176
FoV read	256 mm
FoV phase	93.8 %
Slice thickness	1.00 mm
TR	2300.0 ms
TE	2.98 ms
Averages	1
Concatenations	1
Filter	Distortion Corr.(3D), Prescan Normalize
Coil elements	HE1-4

Contrast - Common

TR	2300.0 ms
TE	2.98 ms
Magn. preparation	Non-sel. IR
TI	900 ms
Flip angle	9 deg
Fat suppr.	None
Water suppr.	None

Contrast - Dynamic

Averages	1
Averaging mode	Long term
Reconstruction	Magnitude
Measurements	1
Multiple series	Each measurement

Resolution - Common

FoV read	256 mm
FoV phase	93.8 %
Slice thickness	1.00 mm
Base resolution	256
Phase resolution	100 %
Slice resolution	100 %
Phase partial Fourier	Off
Slice partial Fourier	Off

Resolution - Common

Interpolation	Off

Resolution - iPAT

PAT mode	GRAPPA
Accel. factor PE	2
Ref. lines PE	32
Accel. factor 3D	1
Reference scan mode	Integrated

Resolution - Filter Image

Image Filter	Off
Distortion Corr.	On
Mode	3D
Unfiltered images	Off
Prescan Normalize	On
Unfiltered images	Off
Normalize	Off
B1 filter	Off

Resolution - Filter Rawdata

Raw filter	Off
Elliptical filter	Off

Geometry - Common

Slab group	1
Slabs	1
Dist. factor	50 %
Position	Isocenter
Orientation	Sagittal
Phase enc. dir.	A >> P
Slice oversampling	0.0 %
Slices per slab	176
FoV read	256 mm
FoV phase	93.8 %
Slice thickness	1.00 mm
TR	2300.0 ms
Multi-slice mode	Single shot
Series	Interleaved
Concatenations	1

Geometry - AutoAlign

Slab group	1
AutoAlign	Head > Basis
Position	Isocenter
Orientation	Sagittal
Phase enc. dir.	A >> P
Initial Position	Isocenter
L	0.0 mm
P	0.0 mm
H	0.0 mm
Initial Rotation	0.00 deg
Initial Orientation	Sagittal

Geometry - Navigator

System - Miscellaneous

Positioning mode	ISO
Table position	H
Table position	0 mm
MSMA	S - C - T

- 4 -

SIEMENS MAGNETOM Skyra

System - Miscellaneous

Sagittal	R >> L
Coronal	A >> P
Transversal	F >> H
Coil Combine Mode	Adaptive Combine
Save uncombined	Off
Matrix Optimization	Off
AutoAlign	Head > Basis
Coil Select Mode	Off - All

System - Adjustments

B0 Shim mode	Standard
B1 Shim mode	TrueForm
Adjust with body coil	Off
Confirm freq. adjustment	Off
Assume Dominant Fat	Off
Assume Silicone	Off
Adjustment Tolerance	Auto

System - Adjust Volume

Position	Isocenter
Orientation	Sagittal
Rotation	0.00 deg
A >> P	240 mm
F >> H	256 mm
R >> L	176 mm
Reset	Off

System - Tx/Rx

Frequency 1H	123.138297 MHz
Correction factor	1
Gain	Low
Img. Scale Cor.	1.000
Reset	Off
? Ref. amplitude 1H	0.000 V

Physio - Signal1

1st Signal/Mode	None
TR	2300.0 ms
Concatenations	1

Physio - Cardiac

Magn. preparation	Non-sel. IR
TI	900 ms
Fat suppr.	None
Dark blood	Off
FoV read	256 mm
FoV phase	93.8 %
Phase resolution	100 %

Physio - PACE

Resp. control	Off
Concatenations	1

Inline - Common

Subtract	Off
Measurements	1
StdDev	Off
Save original images	On

Inline - MIP

MIP-Sag	Off
MIP-Cor	Off
MIP-Tra	Off

Inline - MIP

MIP-Time	Off
Save original images	On

Inline - Composing

Distortion Corr.	On
Mode	3D
Unfiltered images	Off

Sequence - Part 1

Introduction	On
Dimension	3D
Elliptical scanning	Off
Reordering	Linear
Asymmetric echo	Off
Flow comp.	No
Multi-slice mode	Single shot
Echo spacing	7.1 ms
Bandwidth	240 Hz/Px

Sequence - Part 2

RF pulse type	Fast
Gradient mode	Normal
Excitation	Non-sel.
RF spoiling	On
Incr. Gradient spoiling	Off
Turbo factor	176

Sequence - Assistant

Mode	Off

- 5 -

SIEMENS MAGNETOM Skyra

\\USER\CCF_MS_Brain_MS-PATHS\CCF_Dot_MS_Brain_Project\protocol\3D FLAIR - MS-P *
TA: 6:27 PM: FIX Voxel size: 1.0×1.0×1.0 mmPAT: 2 Rel. SNR: 1.00 : spcir

Properties

Prio recon	Off
Load images to viewer	On
Inline movie	Off
Auto store images	On
Load images to stamp segments	On
Load images to graphic segments	On
Auto open inline display	Off
Auto close inline display	Off
Start measurement without further preparation	Off
Wait for user to start	Off
Start measurements	Single measurement

Routine

Slab group	1
Slabs	1
Position	Isocenter
Orientation	Sagittal
Phase enc. dir.	A >> P
AutoAlign	Head > Basis
Phase oversampling	0 %
Slice oversampling	0.0 %
Slices per slab	176
FoV read	256 mm
FoV phase	93.8 %
Slice thickness	1.00 mm
TR	5000 ms
TE	393 ms
Averages	1.0
Concatenations	1
Filter	Raw filter, Distortion Corr.(3D), Prescan Normalize
Coil elements	HE1-4

Contrast - Common

TR	5000 ms
TE	393 ms
MTC	Off
Magn. preparation	Non-sel. T2-IR
TI 1	1800 ms
Fat suppr.	None
Blood suppr.	Off
Restore magn.	Off

Contrast - Dynamic

Averages	1.0
Reconstruction	Magnitude
Measurements	1
Multiple series	Each measurement

Resolution - Common

FoV read	256 mm
FoV phase	93.8 %
Slice thickness	1.00 mm
Base resolution	256
Phase resolution	100 %
Slice resolution	100 %
Phase partial Fourier	Allowed
Slice partial Fourier	7/8
Interpolation	Off

Resolution - iPAT

PAT mode	GRAPPA
Accel. factor PE	2
Ref. lines PE	28
Accel. factor 3D	1
Reference scan mode	Integrated

Resolution - Filter Image

Image Filter	Off
Distortion Corr.	On
Mode	3D
Unfiltered images	Off
Prescan Normalize	On
Unfiltered images	Off
Normalize	Off
B1 filter	Off

Resolution - Filter Rawdata

Raw filter	On
Elliptical filter	Off

Geometry - Common

Slab group	1
Slabs	1
Position	Isocenter
Orientation	Sagittal
Phase enc. dir.	A >> P
Slice oversampling	0.0 %
Slices per slab	176
FoV read	256 mm
FoV phase	93.8 %
Slice thickness	1.00 mm
TR	5000 ms
Series	Interleaved
Concatenations	1

Geometry - AutoAlign

Slab group	1
AutoAlign	Head > Basis
Position	Isocenter
Orientation	Sagittal
Phase enc. dir.	A >> P
Initial Position	Isocenter
L	0.0 mm
P	0.0 mm
H	0.0 mm
Initial Rotation	0.00 deg
Initial Orientation	Sagittal

Geometry - Saturation

Fat suppr.	None
Restore magn.	Off
Special sat.	None

Geometry - Navigator

System - Miscellaneous

Positioning mode	FIX
Table position	H
Table position	0 mm
MSMA	S - C - T

- 6 -

SIEMENS MAGNETOM Skyra

System - Miscellaneous

Sagittal	R >> L
Coronal	A >> P
Transversal	F >> H
Coil Combine Mode	Adaptive Combine
Save uncombined	Off
Matrix Optimization	Off
AutoAlign	Head > Basis
Coil Select Mode	Off - All

System - Adjustments

B0 Shim mode	Standard
B1 Shim mode	TrueForm
Adjust with body coil	Off
Confirm freq. adjustment	Off
Assume Dominant Fat	Off
Assume Silicone	Off
Adjustment Tolerance	Auto

System - Adjust Volume

Position	Isocenter
Orientation	Sagittal
Rotation	0.00 deg
A >> P	240 mm
F >> H	256 mm
R >> L	176 mm
Reset	Off

System - Tx/Rx

Frequency 1H	123.138297 MHz
Correction factor	1
Gain	High
Img. Scale Cor.	1.000
Reset	Off
? Ref. amplitude 1H	0.000 V

Physio - Signal1

1st Signal/Mode	None
Trigger delay	0 ms
TR	5000 ms
Concatenations	1

Physio - Cardiac

Magn. preparation	Non-sel. T2-IR
TI 1	1800 ms
Fat suppr.	None
Dark blood	Off
FoV read	256 mm
FoV phase	93.8 %
Phase resolution	100 %

Physio - PACE

Resp. control	Off
Concatenations	1

Inline - Common

Subtract	Off
Measurements	1
StdDev	Off
Save original images	On

Inline - MIP

MIP-Sag	Off
MIP-Cor	Off
MIP-Tra	Off
MIP-Time	Off
Save original images	On

Inline - Composing

Distortion Corr.	On
Mode	3D
Unfiltered images	Off

Sequence - Part 1

Introduction	On
Dimension	3D
Elliptical scanning	Off
Reordering	Linear
Flow comp.	No
Echo spacing	3.42 ms
Adiabatic-mode	Off
Bandwidth	781 Hz/Px

Sequence - Part 2

Echo train duration	852 ms
RF pulse type	Normal
Gradient mode	Fast
Excitation	Non-sel.
Flip angle mode	T2 var
Turbo factor	270

Sequence - Assistant

Allowed delay	30 s

- 7 -

Appendix B – Protocol Set-Up Tips & Common Issues

Following 2 pages.

<table>
<tr><th>Issue</th><th>Software Version(s)</th><th>Note</th></tr>
<tr><td>Interpolation</td><td>ALL</td><td>Interpolation should be set to “OFF” for both sequences.</td></tr>
<tr><td>Prescan Normalize</td><td>ALL</td><td>“Prescan normalize” filter should be set to “ON” for both sequences.</td></tr>
<tr><td rowspan="2">Distortion Correction</td><td>Prior to B19</td><td>T1: use in-line 3D distortion correction.

FLAIR: in-line 3D distortion correction for the SPACE sequence is only available for software versions B19 onwards.
Workaround:
• Leave in-line distortion correction OFF.
• After acquisition – from Patient Browser, select FLAIR series > “Evaluation” > “3D Distortion Correction”
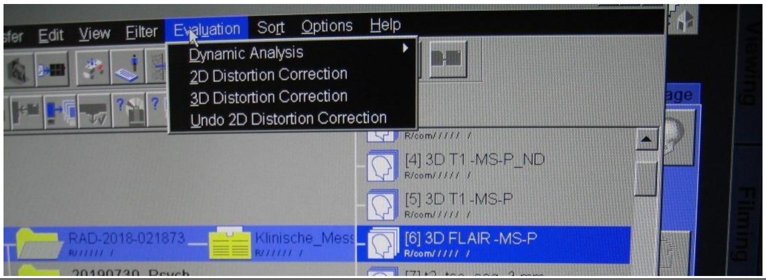
</td></tr>
<tr><td>B19 and later</td><td>T1 & FLAIR: use in-line 3D distortion correction.</td></tr>
<tr><td>Morpho Add-In</td><td>Prior to VE11</td><td>Include the “Morpho” add-in for the MPRAGE (drag and drop from the Dot Cockpit program editor):</td></tr>
</table>

<table>
<tr><td></td><td></td><td>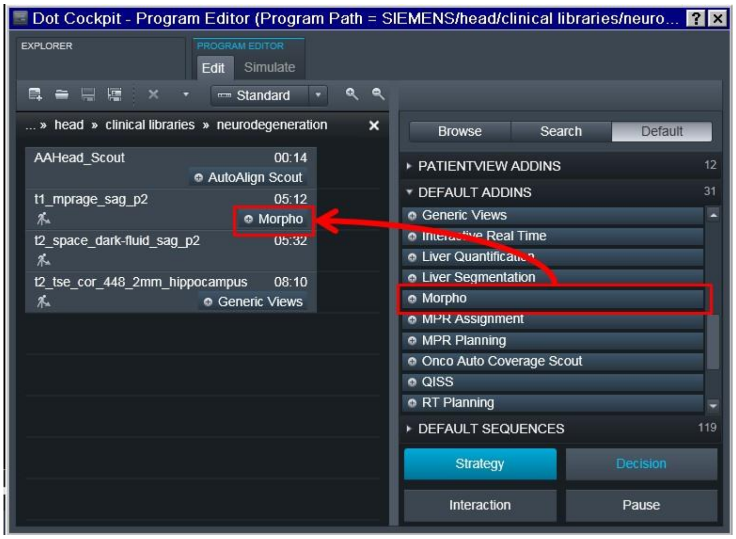
</td></tr>
</table>

<table>
<tr><th>Issue</th><th>Software Version(s)</th><th>Note</th></tr>
<tr><td>Coil Select Mode</td><td>ALL</td><td>We recommend deactivating automatic coil selection, as body/spine/neck coil selection can cause intensity normalization issues.

Step 1 – select only head coils (“System” > “Coils” card):
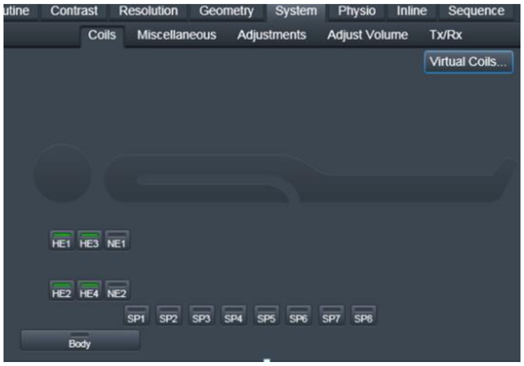

Step 2 – set “Coil Select Mode” to “Off – All” (“System” > “Miscellaneous” card):
</td></tr>
</table>

		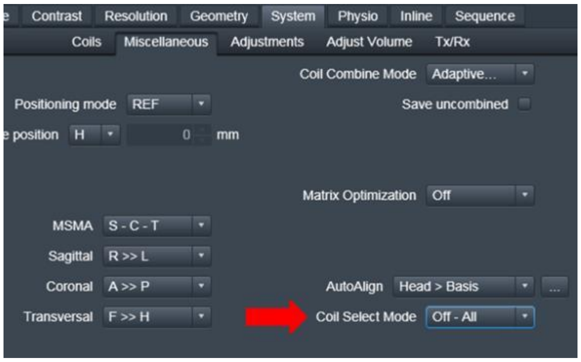
**Series Descriptions**	ALL	Specific series descriptions are <u>required for automated data transfer.</u> <u>**"T1" or "FLAIR" and the "MS-P" tag must be present.**</u> These are the preferred series descriptions:<br>• "3D-T1-MS-P"<br>• "3D-FLAIR-MS-P"<br>If different series descriptions are required at your institution, please work with the MS PATHS imaging team to ensure data transfer will still occur as expected.

Appendix C – Artifacts

Figure 1. Motion in a 3D FLAIR can

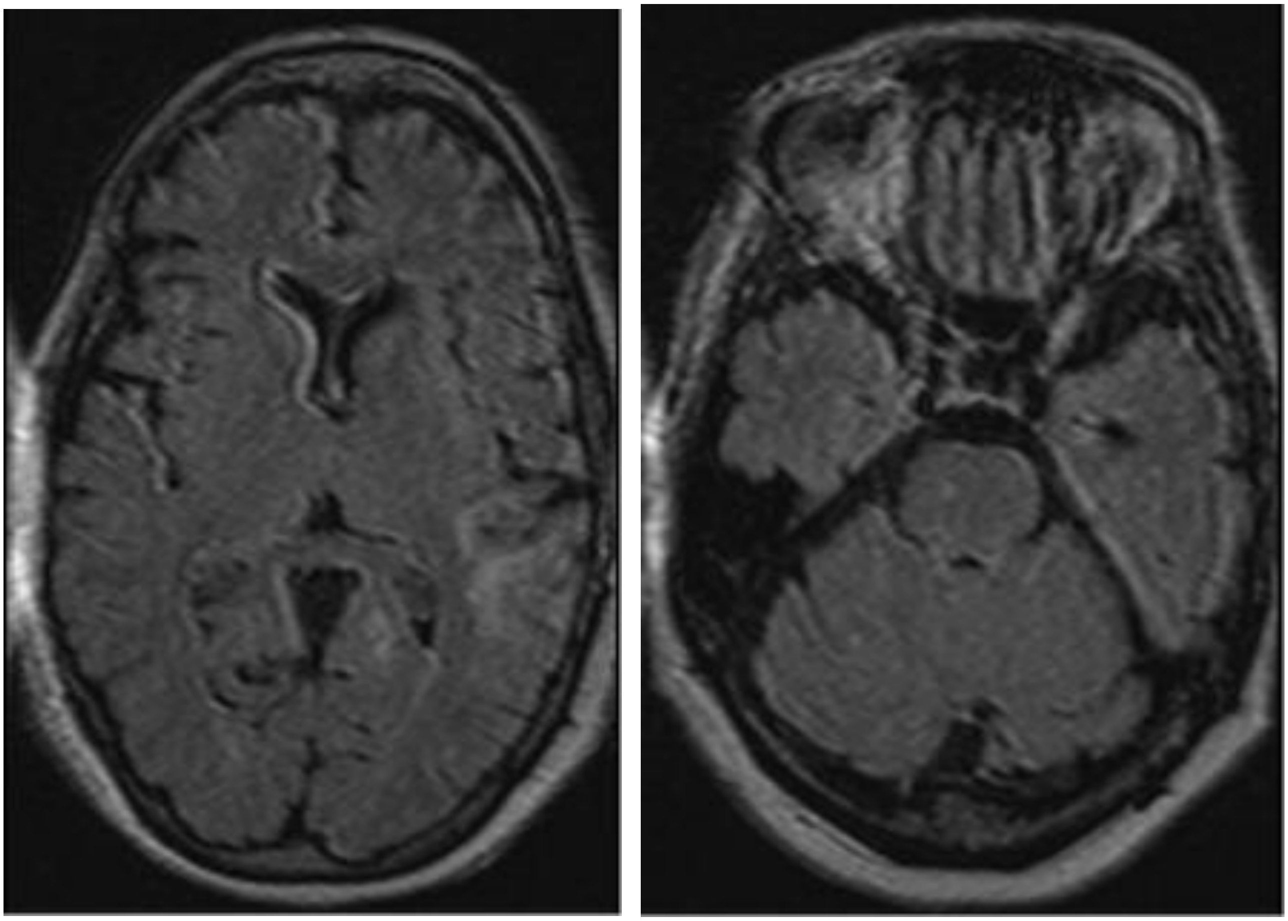

Figure 2. Insufficient nulling of the CSF

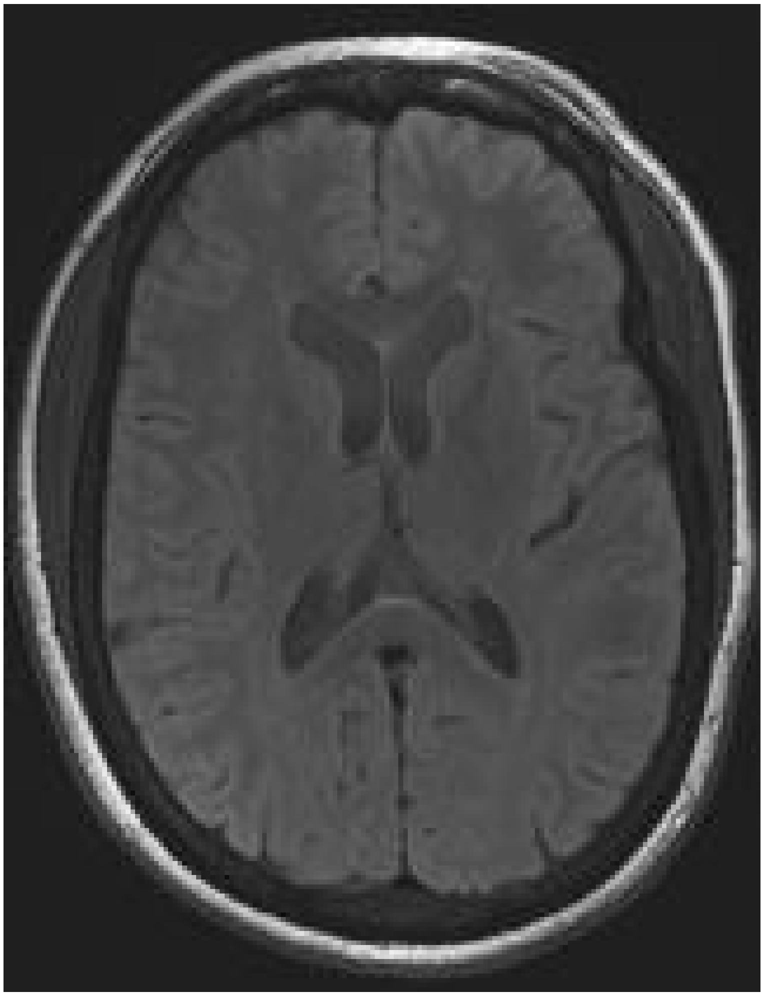

Figure 3. Prescan normalize artifact

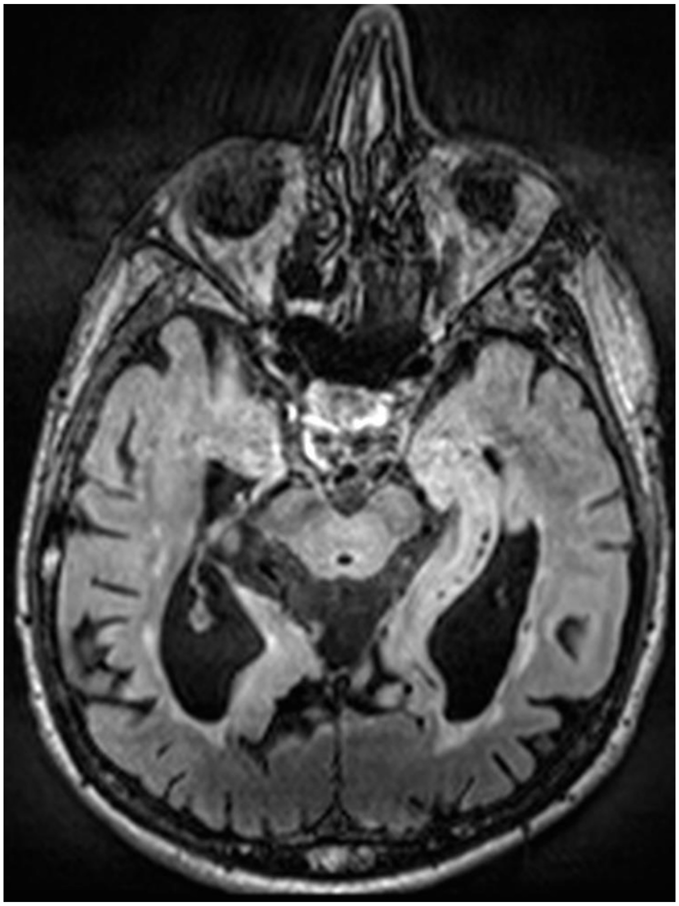

Appendix D – Quality Review Form

Following 2 pages.

MS PATHS
Partners Advancing Technology and Health Solutions

MS PATHS MRI QUALITY FORM – 888MS001

MS-PATHS Global ID:	Acquisition Date:		Scanner:
	Yes	No	Comments/Description of Problem
Scanner change or upgrade?	☐	☐	
Complete brain coverage?	☐	☐	
MPRAGE acquired pre-Gd?	☐	☐	

Image Quality Review

FLAIR			MPRAGE		
Fail	Evaluation	Comments	Fail	Parameter	Comments
☐	SNR		☐	SNR	
☐	Brain:CSF CNR		☐	Brain:CSF CNR	
☐	NAWM:Lesion CNR		☐	NAWM:Lesion CNR	

Protocol Compliance

FLAIR				MPRAGE			
Fail	Parameter	Value	Reference	Fail	Parameter	Value	Reference
☐	Orientation		Sagittal	☐	Orientation		Sagittal
☐	MR Acquisition Type		3D	☐	MR Acquisition Type		3D
☐	Repetition Time (ms)		5000	☐	Repetition Time (ms)		2300
☐	Echo Time (ms)		392 - 394	☐	Echo Time (ms)		2.96 - 2.98
☐	Inversion Time (ms)		1800	☐	Inversion Time (ms)		900
☐	Read FOV (mm)		256	☐	Read FOV (mm)		256
☐	Phase FOV (mm)		240	☐	Phase FOV (mm)		240
☐	Percent Phase FoV		93.75 - 100%	☐	Percent Phase FoV		93.75 - 100%
☐	Phase Encoding Direction		A >> P	☐	Phase Encoding Direction		A >> P
☐	Slice Thickness (mm)		1	☐	Slice Thickness (mm)		1
☐	Pixel Spacing (mm x mm)		1 x 1	☐	Pixel Spacing (mm x mm)		1 x 1
☐	Pixel Bandwidth (Hz/Px)		780 - 781	☐	Pixel Bandwidth (Hz/Px)		240
☐	Interpolation		OFF	☐	Interpolation		OFF
☐	Distortion Correction		ON / 3D mode	☐	Distortion Correction		ON / 3D mode

MS PATHS
Partners Advancing Technology and Health Solutions

MS PATHS MRI QUALITY FORM – 888MS001

Image Artifact Review

**FLAIR**					
**Artifact**	**N/A**	**Mild**	**Moderate**	**Severe**	**Comments**
Motion	☐	☐	☐	☐	
Aliasing	☐	☐	☐	☐	
Flow	☐	☐	☐	☐	
Dental/Other susceptibility artifact	☐	☐	☐	☐	
**MPRAGE**					
**Artifact**	**N/A**	**Mild**	**Moderate**	**Severe**	**Comments**
Motion	☐	☐	☐	☐	
Aliasing	☐	☐	☐	☐	
Flow	☐	☐	☐	☐	
Dental/Other susceptibility artifact	☐	☐	☐	☐	

		**FLAIR**	**MPRAGE**
☐ **PASS**			
☐ **FAIL**	Incorrect sequence	☐	☐
	Incomplete brain coverage	☐	☐
	Insufficient image quality (incl. severe artifacts)	☐	☐

Date Received: **Date Reviewed:** **Initials of Reviewer:**

Comments:

For quality review failures, please contact [redacted] Please include MPID in the subject header of the email.
