# Supplementary material for: Harnessing Real-World Data to Inform Decision-Making: Multiple Sclerosis Partners Advancing Technology and Health Solutions (MS PATHS)
Source: Front Neurol. 2020 Aug 7;11:632. doi: 10.3389/fneur.2020.00632 (PMC7426489; doi:10.3389/fneur.2020.00632)
Supplement: Supplementary file 2 [file Data_Sheet_2.PDF]

Biogen MA Inc.
250 Binney Street
Cambridge, MA 02142
United States

Biogen Idec Research Limited
Innovation House
70 Norden Road
Maidenhead Berkshire
SL6 4AY
United Kingdom

PROTOCOL NUMBER: 888MS002

PHASE OF DEVELOPMENT: Not applicable

PROTOCOL TITLE: Multiple Sclerosis Partners Advancing Technology and Health Solutions (MS PATHS) Biobanking Sub-Study for Future Biomarker and Biologic Research

DATE: 12 August 2019
Version 3.0
FINAL

CONFIDENTIAL
The information contained herein may not be used, disclosed, or published without the written consent of Biogen MA Inc.

SPONSOR SIGNATURE PAGE

TABLE OF CONTENTS

SPONSOR SIGNATURE PAGE 2
1. SPONSOR INFORMATION 6
2. LIST OF ABBREVIATIONS 7
3. SYNOPSIS 8
4. SCHEDULE OF ACTIVITIES FOR STUDY 888MS002 12
5. INTRODUCTION 14
5.1. Overview of Multiple Sclerosis 14
5.2. Overview of Learning Health Systems 14
5.3. Overview of Multiple Sclerosis Partners Advancing Technology and Health Solutions 15
5.4. Study Rationale 16
6. STUDY OBJECTIVE 17
7. STUDY DESIGN 18
7.1. Study Overview 18
7.2. Overall Study Duration and Follow-Up 18
7.2.1. Baseline Visit 18
7.2.2. Follow-Up Visits 18
7.2.3. Unscheduled Visits 18
7.3. Study Stopping Rules 19
7.4. End of Study 20
8. SELECTION OF PATIENTS 21
8.1. Inclusion Criteria 21
8.2. Exclusion Criteria 21
9. ENROLLMENT AND REGISTRATION 22
10. WITHDRAWAL OF PATIENTS FROM THE STUDY 23
11. STUDY PROCEDURES 24
11.1. Blood Collection 24
11.1.1. Patients 18 Years of Age or Older 24
11.1.2. Patients Aged 8 to 17 Years (Inclusive) 25
11.2. Sample Requisition Form 25
12. SAFETY ASSESSMENTS 26

CONFIDENTIAL
The information contained herein may not be used, disclosed, or published without the written consent of Biogen MA Inc.

13. SAFETY DEFINITIONS, RECORDING, REPORTING, AND RESPONSIBILITIES 27
13.1. Definitions 27
13.1.1. Adverse Event 27
13.1.2. Serious Adverse Event 27
13.2. Monitoring and Recording Events 27
13.3. Procedures for Handling Special Situations 28
13.3.1. Medical Emergency 28
13.4. Safety Responsibilities 28
13.4.1. The Investigator 28
13.4.2. Biogen 29
14. STATISTICAL METHODS 30
14.1. Biomarker Analyses/Genomics 30
14.2. Sample Size Considerations 30
15. ETHICAL REQUIREMENTS 31
15.1. Declaration of Helsinki 31
15.2. Institutional Review Board/Ethics Committee 31
15.3. Patient Information and Consent 31
15.4. Patient Data Protection 32
15.5. Compensation for Injury 32
15.6. Conflict of Interest 32
16. ADMINISTRATIVE PROCEDURES 33
16.1. Study Site Initiation 33
16.2. Quality Assurance 33
16.3. Monitoring of the Study 33
16.4. Study Funding 33
16.5. Publications 33
17. FURTHER REQUIREMENTS AND GENERAL INFORMATION 34
17.1. External Contract Organizations 34
17.1.1. Remote Data Capture 34
17.2. Study Committee 34
17.3. Changes to Final Study Protocol 34

CONFIDENTIAL
The information contained herein may not be used, disclosed, or published without the written consent of Biogen MA Inc.

17.4. IRB/Ethics Committee Notification of Study Completion or Termination 35
17.5. Retention of Study Data 35
18. REFERENCES 36
19. SIGNED AGREEMENT OF THE STUDY PROTOCOL 37

LIST OF TABLES

Table 1: Schedule of Activities 12

1. SPONSOR INFORMATION

In North America, Biogen MA Inc. (Cambridge, US) is the Sponsor of the study. In the Rest of World, Biogen Idec Research Limited (Maidenhead, UK) is the Sponsor of the study. Biogen is responsible for initiating and managing the study.

Biogen MA Inc.<br>250 Binney Street<br>Cambridge, MA 02142<br>United States	Biogen Idec Research Limited<br>Innovation House<br>70 Norden Road<br>Maidenhead, Berkshire<br>SL6 4AY<br>United Kingdom

Biogen may transfer any or all of its study-related responsibilities to a contract research organization and other third parties; however, Biogen retains overall accountability for these activities.

2. LIST OF ABBREVIATIONS

AE	adverse event
CNS	central nervous system
DMT	disease-modifying therapy
DNA	deoxyribonucleic acid
GCP	Good Clinical Practice
ICF	informed consent form
ICH	International Council for Harmonisation
IOM	Institute of Medicine
IRB	institutional review board
K_2 EDTA	potassium ethylenediaminetetraacetic acid
LHS	Learning Health System
MS	multiple sclerosis
MS PATHS	Multiple Sclerosis Partners Advancing Technology and Health Solutions
PHI	protected health information
RNA	ribonucleic acid
SABR	Safety and Benefit-Risk Management
SAE	serious adverse event
SRF	sample requisition form
US	United States

CONFIDENTIAL
The information contained herein may not be used, disclosed, or published without the written consent of Biogen MA Inc.

3. SYNOPSIS

Protocol Number:	888MS002
Protocol Title:	Multiple Sclerosis Partners Advancing Technology and Health Solutions (MS PATHS) Biobanking Sub-Study for Future Biomarker and Biologic Research
Version Number	3
Name of Study Treatment:	None
Study Indication:	Multiple sclerosis (MS)
Study Rationale:	This biobanking sub-study will further enhance the goals of the Multiple Sclerosis Partners Advancing Technology and Health Solutions (MS PATHS) demonstration project (henceforth referred to as Study 888MS001) by adding biosamples to the standardized clinical and radiologic data and MS PATHS infrastructure.<br><br>Biomarker and genomic data may uniquely contribute to the understanding of MS disease etiology, pathophysiological mechanisms associated with the disease, and treatment response in MS. For example, characterization of how biomarkers change as the disease process evolves may contribute to an enhanced understanding of MS and its progression. Experience with treatment in MS shows that there is heterogeneity in clinical response, and some of the heterogeneity may be associated with genetic variation in patients. It is postulated that clinical, radiologic, and/or molecular features of a given patient may eventually enable an individualized prognosis regarding risk for subsequent disease worsening as well as inform treatment decisions.
Phase of Development:	Not applicable
Study Objective:	The primary objective of this sub-study is to develop a longitudinal biobank via collection, processing, shipping, and storage of biosamples from patients with a confirmed diagnosis of clinically isolated syndrome or MS. Three major sample types will be collected from all sites under this sub-study: whole blood for deoxyribonucleic acid, ribonucleic acid, and serum.

Study Design:	This is a multi-site longitudinal sub-study of patients with a confirmed diagnosis of clinically isolated syndrome or MS enrolled in Study 888MS001. Blood sample collections for this sub-study will typically be performed during routine clinical visits. Biological samples will be collected from patients, processed (serum samples only) and stored locally in the short term, and then shipped to a central laboratory for processing and storage in the MS PATHS biobank until the sample is requested and approved for a research study. The de-identified biosamples and the data derived from them will be linked to additional de-identified data collected through MS PATHS (i.e., Study 888MS001 and future sub-studies). Data collected from MS PATHS include standardized demographic, clinical, and radiologic data.
Visit Schedule:	The sample collected at enrollment from the patient will typically coincide with a routine clinical visit. After the enrollment, annual Follow-Up Visits will typically coincide with routine clinical visits for the duration of the study but not occur more frequently than 10 months since the last Baseline or Follow-Up Visit. Unscheduled Visits may occur for clinical activity as defined in Section 7.2.3. After an Unscheduled Visit, the next annual Follow-Up Visit should occur no sooner than 10 months later.
Study Location:	Approximately 10 sites in the United States and Europe are planned.
Number of Planned Patients:	The number of patients enrolled in Study 888MS002 will depend on the enrollment rate of Study 888MS001 and the consent rate into this sub-study (888MS002). It is estimated that approximately 9,000 to 25,000 patients will be enrolled in Study 888MS001.
Study Population:	This sub-study will be conducted in patients with a confirmed diagnosis of clinically isolated syndrome or MS.

Inclusion Criteria

To be eligible to participate in this sub-study, candidates must meet the following eligibility criteria at the time of enrollment prior to provision of consent (and assent, if applicable):

1. Be enrolled in MS PATHS under Study 888MS001.

	2. Patient (or patient’s legal representative) has the ability to understand the purpose and risks of this sub-study and provide any required signed and dated informed consent (or assent) and authorization to use protected health information in accordance with national and local patient privacy regulations. Patients under 18 years of age must have the consent and authorization of a parent or legal guardian. Patients under 18 years of age who have the capacity should provide their assent in addition to the parental/guardian consent, as appropriate, per local regulations.<br><br>3. Age ≥8 years.<br><br>**Exclusion Criteria**<br><br>Candidates will be excluded from this sub-study if any of the following exclusion criteria exist at the time of enrollment prior to provision of consent (and assent, if applicable):<br><br>1. Patients under 18 years of age who fall below the minimum weight of 24 kg for the pediatric population will be excluded.<br><br>2. Any patient who does not meet criteria for blood collection by local and/or national standards will be excluded.<br><br>3. Other unspecified reasons that, in the opinion of the Investigator or Biogen, make the patient unsuitable for participation in this sub-study.
Treatment Groups:	Not applicable
Duration of Study Participation:	This biobanking sub-study will run the duration of Study 888MS001.
Withdrawal from Study:	Patients (or their legally authorized representatives) may request to withdraw from this sub-study at any time and for any reason. Patients may also be required to withdraw from this sub-study (e.g., if they withdraw from Study 888MS001). Upon withdrawal from the study, patients (or their legally authorized representatives) can choose to withdraw consent to further use of collected

	samples, and the samples will be destroyed in that case.[1] However, such withdrawal will be prospective, and all data generated under consent (and assent, if applicable) will be retained.
Study Procedures:	As part of this biobanking sub-study, patients will provide consent/assent, register for the study, and have blood drawn. In addition, the study staff will collect the time of draw, time of last meal, smoking status, and reason for Unscheduled Visit (if applicable) on a sample requisition form. Future biomarker and biologic assessments will be performed by individual researchers at Biogen, at the MS PATHS academic institutions, and/or by their collaborators.[2]
Safety Assessments:	Information on serious adverse events related to the venipunctures/blood draws will be collected during this sub-study. No other safety assessments will be performed.
Statistical Statement and Analytical Plan:	The biosamples collected in this sub-study will be available to Biogen, MS PATHS academic institutions, and/or their collaborators[2] for a variety of research questions and statistical analyses that are not yet determined. All analyses will be governed by the principles, rules, and processes developed by the MS PATHS steering committee.
Study Stopping Rules:	Biogen will have the opportunity to limit or stop enrollment based on periodic MS PATHS biobank assessments (e.g., number of available samples). Biogen may terminate this sub-study if proper notice is given to the collaborating health care institutions, as stipulated in the project contracts.
End of Study:	This biobanking sub-study will end at the same time as Study 888MS001.

[1] The provision for destruction will not apply to samples that have already been released from the biobank for research purposes.
[2] The researchers/collaborators may engage third parties to conduct biomarker and biologic assessments.

CONFIDENTIAL
The information contained herein may not be used, disclosed, or published without the written consent of Biogen MA Inc.

4. SCHEDULE OF ACTIVITIES FOR STUDY 888MS002

Table 1: Schedule of Activities

Assessments	Baseline Visit[1]	Follow-Up Visit(s)[2]	Unscheduled Visit(s)[3]
Informed Consent/Assent Form(s)[4, 5]	X		
Enrollment and Registration	X		
Sample Requisition Form[6]	X	X	X
Blood Collection for RNA, Serum, and Whole Blood Biomarker Samples[7,8]	X	X	X
Blood Collection for DNA Sample[9]	X		
SAE Reporting	------Only SAEs related to the venipunctures/blood draws will be collected during this sub-study.------		

DNA = deoxyribonucleic acid; K_2 EDTA = potassium ethylenediaminetetraacetic acid; MS PATHS = Multiple Sclerosis Partners Advancing Technology and Health Solutions; RNA = ribonucleic acid; SAE = serious adverse event.

[1] The Baseline Visit may occur concurrently with enrollment in Study 888MS001 or at any point thereafter prior to the end of that study.

[2] After the Baseline Visit, annual Follow-Up Visits will typically coincide with routine clinical visits for the duration of the study but not occur more frequently than 10 months since the last Baseline or Follow-Up Visit.[3] Unscheduled visits may occur for clinical activity as defined in Section 7.2.3. After an Unscheduled Visit, the next annual Follow-Up Visit should occur no sooner than 10 months later.

[4] The Investigator will verify patient eligibility for study participation prior to signing of the informed consent (and assent, if applicable) form(s).

[5] Written informed consent must be obtained from any patient (or their legally authorized representative) who opts to participate in this biobanking sub-study; an additional written consent must be obtained from patients (or their legally authorized representatives) who elect to provide blood samples for genetic testing. Patients under 18 years of age who have the capacity should provide written assent in additional to the parental/guardian consent. The consent/assent form(s) must be signed and dated before any data collection is performed. Patients may withdraw consent/assent at any time, and no withdrawal visit is required.

[6] The sample requisition form will document the following information: date and time of sample collection, time of last meal, smoking status, and reason for unscheduled visit (if applicable).

[7] In patients ≥18 years of age, blood collection for future biomarker assessments will typically occur during routine clinical visits but not more frequently than every 10 months. Blood collection for these samples will total 28 mL whole blood: 5 mL into 2 PAXgene RNA tubes (2.5 mL each) for RNA, 17 mL into 2 SST™ tubes (8.5 mL each) for serum, and 6 mL into one 6-mL K_2 EDTA tube for epigenetic and other whole blood biomarkers.

[8] In patients 8 to 17 years of age (inclusive), blood collection for future biomarker assessments will typically occur during routine clinical visits but not more frequently than every 10 months. Blood collection for these samples will total 13.5 mL whole blood: 5 mL into 2 PAXgene RNA tubes (2.5 mL each) for RNA and 8.5 mL into one 8.5-mL SST™ tube for serum.

CONFIDENTIAL
The information contained herein may not be used, disclosed, or published without the written consent of Biogen MA Inc.

[9] In patients ≥18 years of age, blood collection for DNA samples will total 10 mL whole blood into one 10-mL K_2 EDTA tube for genetic testing. In patients 8 to 17 years of age (inclusive), blood collection for DNA samples will total 6 mL whole blood into one 6-mL K_2 EDTA tube for genetic testing. For all patients, collection of the genetic testing sample is preferred at the Baseline Visit but may be completed at any single visit during study participation.

Note: All blood collection volumes may vary slightly across sites based on tubes available in different countries.

CONFIDENTIAL
The information contained herein may not be used, disclosed, or published without the written consent of Biogen MA Inc.

5. INTRODUCTION

5.1. Overview of Multiple Sclerosis

Multiple sclerosis (MS) is a chronic inflammatory demyelinating disease of the central nervous system (CNS) that affects approximately 400,000 persons in North America and 365,000 persons in Europe. It is predominantly a disease of young adults, primarily women, with disease onset typically occurring between the ages of 20 and 40 years. MS is the most common form of inflammatory demyelinating CNS disease and the most common cause of nontraumatic neurological disability in young adults. Disease progression and advancing disability are common in patients with MS who are followed for several years.

MS is a heterogeneous disease that can affect a wide range of functional systems, resulting in symptoms that vary from one patient to another. More importantly, the MS spectrum can vary in severity from an active and aggressive disease course from the onset to a mild disease with infrequent relapses [Confavreux 2000]. While early management of MS disease activity is recognized as important for preventing relapses and long-term disability, early intervention in and of itself does not necessarily assure an optimal outcome given the heterogeneous responses to treatment that have been observed.

Information relating to the pathophysiological basis of MS has the potential to further enhance our understanding of the disease and to make possible a personalized patient medicine approach. Genetic, genomic, proteomic, and other existing biomarker data indicate that there are multiple genotypes and clinical phenotypes underlying the disease collectively referred to as MS [Achiron 2012; De Jager 2011; Hecker 2011; Martinelli-Boneschi 2012; Ottoboni 2012; Vecchio 2011]. It is hypothesized that patients with different disease phenotypes will not only exhibit different disease courses but also will respond differently to a given treatment, leading to inter-individual differences in clinical response. It is postulated that clinical, radiologic, and/or molecular features of a given patient may eventually enable an individualized prognosis regarding risk for subsequent disease worsening as well as inform treatment decisions.

5.2. Overview of Learning Health Systems

The Institute of Medicine (IOM) issued a report in 2013 describing the concept of a Learning Health System (LHS) that capitalizes on the existing patient data that flow through the health care system to advance clinical knowledge with every patient visit [IOM 2013]. The report outlined the necessity to evolve such a system to achieve greater value in health care for all stakeholders. The American health care system has been struggling to achieve a level of consistent quality, efficiency, and safety across the nation that, if achieved, could save many lives. Paradoxes in the health care system have held back this advancement of care and value for a multitude of reasons. Although the IOM report is focused on the American health system, many health systems around the globe face similar challenges. First, the coexistence of over-treatment and under-treatment can leave patients with either unnecessary and expensive care or a lack of adequate care due to an absence of standard evidence-based protocols. Second,

CONFIDENTIAL
The information contained herein may not be used, disclosed, or published without the written consent of Biogen MA Inc.

patients' health challenges and the health care system are becoming increasingly complex. As the population grows older, patients are developing more comorbid diseases, but treatment guidelines have not adapted to handle this challenge. Third, the American health system's understanding of diseases is expanding, but with the expansion of medical knowledge comes increasingly complex disease sub-types. Finally, new drugs and devices are being increasingly introduced, but evidence to support identifying the most effective drugs for various disease sub-types continues to lag behind. To move health care forward and establish evidence-based guidelines, an LHS needs to be formed.

The crux of an LHS is aggregation of data from routine clinical practice that can be subsequently used to generate knowledge and insights to improve patient care. The IOM lays out 4 important steps to achieving this LHS. First is generating and using real-time knowledge to improve outcomes. The second key step to achieving an LHS is creating a "culture of care" that supports the vision of continuous improvement with each patient visit. Physicians face pressures from time, chaotic environments, inefficient workflows, administrative burdens, and uncoordinated systems that can distract initiatives that focus on improving care. Having strong leadership and governance that incentivize systematic problem solving, experimentation, and learning from past experiences is essential to minimizing these distractions and focusing on advancement of clinical knowledge. The third step is engaging families, patients, and communities to improve communication and create a truly patient-centered care system. Engaging patients in their own care decisions allows them to give a personal perspective on the suitability of treatments, which can ultimately result in more effective care for the patient. Additionally, involving the patient by collecting self-reported outcomes can help engage them and expand the knowledge base without adding burden to the clinician. Finally, the fourth step is achieving and then rewarding high value care through payment models that focus on value instead of individual services and products. Transparency in payment will help patients choose appropriate providers, which will, in turn, encourage providers and organizations to achieve higher value. These 4 steps can help create a true LHS, where clinicians, researchers, and, ultimately, patients will benefit from a focus on learning through data.

The MS population would especially benefit from such a system, as treatment protocols for MS are less standardized in comparison to other disease states, and few evidence-based treatment guidelines exist to indicate which patients will respond best to the numerous therapies on the market.

5.3. Overview of Multiple Sclerosis Partners Advancing Technology and Health Solutions

The parent study is Multiple Sclerosis Partners Advancing Technology and Health Solutions (MS PATHS), which is a demonstration project of an LHS in MS (the demonstration project is henceforth referred to as Study 888MS001). Study 888MS001 and its sub-studies are being launched by Biogen in collaboration with select health care institutions.

This demonstration project will specifically focus on leveraging technology to generate and aggregate de-identified data from routine care visits across multiple institutions. The goal is to

CONFIDENTIAL
The information contained herein may not be used, disclosed, or published without the written consent of Biogen MA Inc.

ultimately improve outcomes in MS by enabling real-world, broad-scale MS phenotyping that is needed to better understand MS, predict therapeutic responses, and develop meaningful approaches to personalized medicine.

MS PATHS aims to engage all providers and nearly all patients with a confirmed diagnosis of clinically isolated syndrome or MS (henceforth referred to as MS patients) in an MS center to standardize, quantify, and maximize data collected as part of routine care. The demonstration project plans to leverage existing and developing technology with the goal to reduce the data collection burden on health care providers and staff (e.g., Multiple Sclerosis Performance Test device). In addition, the demonstration project will include separately consented sub-studies to collect data outside of routine care. This infrastructure aims to enable researchers to access a large set of longitudinal patient data to advance the knowledge of MS disease progression and patients' response to therapies.

5.4. Study Rationale

This biobanking sub-study will further enhance the goals of Study 888MS001 by adding biosamples to the standardized clinical and radiologic data and MS PATHS infrastructure.

Biomarker and genomic data may uniquely contribute to the understanding of disease etiology, pathophysiological mechanisms associated with the disease, and treatment response in MS. For example, characterization of how biomarkers change as the disease process evolves may contribute to an enhanced understanding of MS and its progression. Experience with treatment in MS shows that there is heterogeneity in clinical response, and some of the heterogeneity may be associated with genetic variation in patients. It is postulated that clinical, radiologic, and/or molecular features of a given patient may eventually enable an individualized prognosis regarding risk for subsequent disease worsening as well as inform treatment decisions.

CONFIDENTIAL
The information contained herein may not be used, disclosed, or published without the written consent of Biogen MA Inc.

6. STUDY OBJECTIVE

The primary objective of this sub-study is to develop a longitudinal biobank via collection, processing, shipping, and storage of biosamples from MS patients. Three major sample types will be collected from all sites under this sub-study: whole blood for deoxyribonucleic acid (DNA), ribonucleic acid (RNA), and serum.

CONFIDENTIAL
The information contained herein may not be used, disclosed, or published without the written consent of Biogen MA Inc.

7. STUDY DESIGN

7.1. Study Overview

This is a multi-site longitudinal sub-study of MS patients enrolled in Study 888MS001. Blood sample collections will typically be performed during routine clinical visits. Biological samples will be collected from patients, processed (serum samples only) and stored locally in the short term, and then shipped to a central laboratory for processing and storage in the MS PATHS biobank until the sample is requested and approved for a research study. The biosamples and the data derived from them will be linked to additional de-identified data collected through MS PATHS (i.e., Study 888MS001 and future sub-studies). Data collected from MS PATHS include standardized demographic, clinical, and radiologic data.

This sub-study is planned to be conducted at approximately 10 sites in the United States (US) and Europe. The number of patients enrolled in Study 888MS002 will depend on the enrollment rate of Study 888MS001 and the consent rate into this sub-study (888MS002). It is estimated that approximately 9,000 to 25,000 patients will be enrolled in Study 888MS001.

7.2. Overall Study Duration and Follow-Up

This biobanking sub-study will continue for the duration of Study 888MS001

The sub-study will consist of the Baseline and Follow-Up Visits.

7.2.1. Baseline Visit

The Baseline Visit will typically coincide with a routine clinical visit. Patient eligibility for the study will be determined at the Baseline Visit prior to obtaining any required consent/assent from the patient and/or the patient's legally authorized representative.

7.2.2. Follow-Up Visits

After the Baseline Visit, annual Follow-Up Visits will typically coincide with routine clinical visits for the duration of the study but not occur more frequently than 10 months since the last Baseline or Follow-Up Visit.

7.2.3. Unscheduled Visits

Unscheduled Visits will be permitted for the following events as determined by the Investigator:

1. Relapse

    a. For the purposes of this study, relapses are defined as new or recurrent neurologic symptoms not associated with fever, lasting at least 24 hours. New or recurrent neurologic symptoms that evolve gradually over months are to be considered disease progression, not an acute

relapse. New or recurrent neurologic symptoms that occur fewer than 30 days following the onset of a relapse as defined above are to be considered part of the same relapse. An Unscheduled Visit for a relapse should occur within 30 days of the relapse onset.

2. MRI activity

    a. For the purposes of this study, MRI activity is any new or enlarging T2 lesion, gadolinium enhancing lesion, or brain atrophy. MRI activity can be based on any MRI (i.e., the MRI does not have to be from a Siemens 3T scanner using the MS PATHS 3D FLAIR and 3D MP-RAGE acquisition sequences). An Unscheduled Visit for MRI activity should occur within 30 days of the MRI acquisition.

3. MS PATHS MRI obtained

    a. For the purposes of this study, a MS PATHS MRI is an MRI acquired on a Siemens 3T scanner using the MS PATHS 3D FLAIR and 3D MP-RAGE acquisition sequences. An Unscheduled Visit for a new MS PATHS MRI should occur within 30 days of MRI acquisition.

4. Disease-modifying therapy (DMT) switch

    a. For the purposes of this study, a DMT switch is the initiation of a new therapy which has an approved indication in the United States or European Union for the treatment of MS or sub-type of MS. Therapies indicated solely for symptomatic treatment are excluded. An Unscheduled Visit for a DMT switch should occur within 30 days of the first administration of the new therapy.

5. Disease-modifying therapy (DMT) switch follow-up

    a. An Unscheduled Visit for a DMT switch follow-up should occur within 90-240 days after the first administration of the new DMT.

After an Unscheduled Visit, the next annual Follow-Up Visit should occur no sooner than 10 months later.

7.3. Study Stopping Rules

Biogen will have the opportunity to limit or stop enrollment based on periodic MS PATHS biobank assessments (e.g., number of available samples). Biogen may terminate this sub-study if proper notice is given to the collaborating health care institutions, as stipulated in the project contracts.

CONFIDENTIAL
The information contained herein may not be used, disclosed, or published without the written consent of Biogen MA Inc.

7.4. End of Study

This biobanking sub-study will end at the same time as Study 888MS001.

8. SELECTION OF PATIENTS

8.1. Inclusion Criteria

To be eligible to participate in this sub-study, candidates must meet the following eligibility criteria at the time of enrollment prior to provision of consent (and assent, if applicable)

1. Be enrolled in MS PATHS under Study 888MS001.

2. Patient (or patient's legally authorized representative) has the ability to understand the purpose and risks of this sub-study and provide any required signed and dated informed consent and authorization to use protected health information (PHI) in accordance with national and local patient privacy regulations. Patients under 18 years of age must have the consent and authorization of a parent or legal guardian. Patients under 18 years of age who have the capacity should provide their assent in addition to the parental/guardian consent, as appropriate, per local regulations.

3. Age ≥8 years.

8.2. Exclusion Criteria

Candidates will be excluded from this sub-study if any of the following exclusion criteria exist at the time of enrollment prior to provision of consent (and assent, if applicable):

1. Patients under 18 years of age who fall below the minimum weight of 24 kg for the pediatric population will be excluded.

2. Any patient who does not meet criteria for blood collection by local and/or national standards will be excluded.

3. Other unspecified reasons that, in the opinion of the Investigator or Biogen, make the patient unsuitable for participation in this sub-study.

CONFIDENTIAL
The information contained herein may not be used, disclosed, or published without the written consent of Biogen MA Inc.

9. ENROLLMENT AND REGISTRATION

The Investigator will verify that a patient is eligible per the criteria in Sections 8.1 and 8.2 prior to the patient (or their legally authorized representative) signing the informed consent form(s) (ICF). Adult patients (or their legally authorized representatives) must provide written informed consent. Patients under 18 years of age must have the consent and authorization of a parent or legal guardian. Patients under 18 years of age who have the capacity should sign a written assent in addition to the parental/guardian consent, as appropriate, per local regulations.

After the provision of consent (and assent, if applicable), the patient is considered to be enrolled in this sub-study and is subsequently registered.

CONFIDENTIAL
The information contained herein may not be used, disclosed, or published without the written consent of Biogen MA Inc.

10. WITHDRAWAL OF PATIENTS FROM THE STUDY

Patients (or their legally authorized representatives) may request to withdraw from this sub-study at any time and for any reason.

Patients will be required to withdraw from this sub-study for any of the following reasons:

- Withdrawal (for any reason) from the parent study, Study 888MS001.
- Unspecified reasons that, in the opinion of the Investigator or Biogen, make the patient unsuitable for continued participation.

Upon withdrawal from the study, patients (or their legally authorized representatives) can choose to withdraw consent to further use of collected samples, and the samples will be destroyed in that case.[3] However, such withdrawal will be prospective, and all data generated under consent (and assent, if applicable) will be retained.

The date and reason for the patient's withdrawal from the study must be recorded in the patient's medical record and the consent management system.

[3] The provision for destruction will not apply to samples that have already been released from the biobank for research purposes.

11. STUDY PROCEDURES

See Section 4 for the timing of all study procedures.

As part of this biobanking sub-study, patients will provide consent/assent, register for the study, and have blood drawn. In addition, the study staff will complete a sample requisition form (SRF) per Section 11.2.

Future biomarker and biologic assessments will be performed by individual researchers at Biogen, at the MS PATHS academic institutions, and/or by their collaborators.[4]

11.1. Blood Collection

Venous blood will be drawn by a trained phlebotomist, registered nurse, or other qualified personnel following written procedures and using standard equipment such as Vacutainer™ brand tubes. No medications will be administered as part of this procedure.

Serum samples will be processed at the study site, and all blood samples will be stored locally in the short term and then shipped to a central laboratory for processing and storage. For detailed procedures related to sample collection, processing, shipping, and storage, please refer to the Laboratory Manual.

11.1.1. Patients 18 Years of Age or Older

At each visit, blood collection for future biomarker assessments will be performed. Blood collection for these samples in patients ≥18 years of age will total 28 mL whole blood, as follows:

- 5 mL into 2 PAXgene RNA tubes (2.5 mL each) for RNA
- 17 mL into 2 SST™ tubes (8.5 mL each) for serum
- 6 mL into one 6-mL potassium ethylenediaminetetraacetic acid (K_2 EDTA) tube for epigenetic and other whole blood biomarkers

Volumes may vary slightly across sites based on tubes available in different countries.

At a single visit, blood collection for future genetic assessments will be performed for those adult patients who consented (or whose legally authorized representatives consented) to the genetic testing component of this sub-study. Collection of the genetic testing sample is preferred at the Baseline Visit but may be completed at any single visit during study participation.

Blood collection for DNA samples in patients ≥18 years of age will total 10 mL whole blood, as follows:

[4] The researchers/collaborators may engage third parties to conduct biomarker and biologic assessments.

- 10 mL into one 10-mL K_2 EDTA tube.

Volumes may vary slightly across sites based on tubes available in different countries.

11.1.2. Patients Aged 8 to 17 Years (Inclusive)

At each visit, blood collection for future biomarker assessments will be performed. Blood collection for these samples in patients 8 to 17 years of age (inclusive) will total 13.5 mL whole blood, as follows:

- 5 mL into 2 PAXgene RNA tubes (2.5 mL each) for RNA
- 8.5 mL into one 8.5-mL SST tube for serum

Volumes may vary slightly across sites based on tubes available in different countries.

At a single visit, blood collection for future genetic assessments will be performed for those pediatric patients whose legally authorized representatives consented to the genetic testing component of this sub-study and who themselves assented, if applicable. Collection of the genetic testing sample is preferred at the Baseline Visit but may be completed at any single visit during study participation. Blood collection for DNA samples in patients 8 to 17 years of age (inclusive) will total 6 mL whole blood, as follows:

- 6 mL into one 6-mL K_2 EDTA tube.

Volumes may vary slightly across sites based on tubes available in different countries.

If an enrolled patient turns 18 years of age during study participation, the patient will be reconsented and will continue participation using blood collection volumes for patients ≥18 years of age as described in Section 11.1.1.

11.2. Sample Requisition Form

At each visit at which blood is collected, the study staff will complete an SRF that includes the following information:

- time of blood collection
- time of last meal
- smoking status
- reason for Unscheduled Visit (if applicable)

12. SAFETY ASSESSMENTS

Information on serious adverse events (SAEs) related to the venipunctures/blood draws will be collected during this sub-study. No other safety assessments will be performed.

CONFIDENTIAL
The information contained herein may not be used, disclosed, or published without the written consent of Biogen MA Inc.

13. SAFETY DEFINITIONS, RECORDING, REPORTING, AND RESPONSIBILITIES

13.1. Definitions

13.1.1. Adverse Event

An adverse event (AE) is any untoward medical occurrence in a patient or clinical investigation subject. An AE can therefore be any unfavorable and unintended sign (including an abnormal laboratory finding), symptom, or disease temporally associated with the patient's participation in the research, whether or not related to the patient's participation in the research.

13.1.2. Serious Adverse Event

An SAE is any untoward medical occurrence that meets any of the following criteria:

- Results in death
- In the view of the Investigator, places the patient at immediate risk of death (a life-threatening event); however, this does not include an event that, had it occurred in a more severe form, might have caused death
- Requires inpatient hospitalization or prolongation of existing hospitalization
- Results in persistent or significant disability/incapacity
- Results in a congenital anomaly/birth defect

An SAE may also be any other medically important event that, in the opinion of the Investigator, may jeopardize the patient or may require intervention to prevent one of the other outcomes listed in the definition above. (Examples of such medical events include allergic bronchospasm requiring intensive treatment in an emergency room or convulsions occurring at home that do not require an inpatient hospitalization.)

13.2. Monitoring and Recording Events

Any SAE related to the venipunctures/blood draws experienced by the patient between the time of the signing of the ICF and the end of this sub-study must be reported to Biogen Safety and Benefit-Risk Management (SABR) or designee within 24 hours of the study site staff becoming aware of the event. To report initial or follow-up information on an SAE related to the venipunctures/blood draws, complete the SAE form and fax or email it to Biogen SABR or designee. Refer to the Investigator Site File for the fax number and email address.

All other AEs should be reported via standard post-marketing channels as appropriate.

CONFIDENTIAL
The information contained herein may not be used, disclosed, or published without the written consent of Biogen MA Inc.

Any SAE related to the venipunctures/blood draws that is ongoing when the patient completes or discontinues this sub-study will be followed by the Investigator until the event has resolved, stabilized, or returned to baseline status.

13.3. Procedures for Handling Special Situations

13.3.1. Medical Emergency

In a medical emergency requiring immediate attention, study site staff will apply appropriate medical intervention, according to current standards of care.

13.4. Safety Responsibilities

13.4.1. The Investigator

The Investigator's responsibilities include the following:

- Review all AEs related to the venipunctures/blood draws to determine seriousness.
- Monitor and record all SAEs related to the venipunctures/blood draws.
- Determine the onset and resolution dates of each SAE related to the venipunctures/blood draws.
- Complete an SAE form for each SAE related to the venipunctures/blood draws and fax or email it to Biogen SABR or designee within 24 hours of the study site staff becoming aware of the event.
- Ensure all SAE reports are supported by documentation in the patients' medical records.
- Report SAEs related to the venipunctures/blood draws to the institutional review board (IRB) or ethics committees, as required by local law.
- Pursue follow-up information actively and persistently for SAEs related to the venipunctures/blood draws. Follow-up information must be reported to Biogen SABR or designee within 24 hours of the study site staff becoming aware of new information.
- Report any AEs (including SAEs) related to patient treatment via standard postmarketing channels as appropriate.

CONFIDENTIAL
The information contained herein may not be used, disclosed, or published without the written consent of Biogen MA Inc.

13.4.2. Biogen

Biogen's responsibilities include the following:

- Before study site activation and patient enrollment, Biogen is responsible for reviewing with study site staff the definitions of an SAE related to venipunctures/blood draws, as well as the instructions for monitoring, recording, and reporting said SAEs.

- Biogen is to notify all appropriate regulatory authorities, central ethics committees, and Investigators of SAEs related to venipunctures/blood draws, as required by local law, within required time frames.

CONFIDENTIAL
The information contained herein may not be used, disclosed, or published without the written consent of Biogen MA Inc.

14. STATISTICAL METHODS

14.1. Biomarker Analyses/Genomics

The biosamples collected through the procedures described in this protocol will be available to Biogen, MS PATHS academic institutions, and/or their collaborators[5] for a variety of research questions and statistical analyses that are not yet determined. Biologic samples and data collected on the SRF will be linked to additional de-identified data collected through MS PATHS studies (i.e., Study 888MS001 and future sub-studies), including clinical and radiologic data. All analyses will be governed by the principles, rules, and processes developed by the MS PATHS steering committee.

Biological samples will be used to study MS disease characteristics, pharmacodynamic responses, and treatment-related changes and for future scientific research in MS and other medical conditions. Multitudes of sample and data analyses can be envisioned based on technological advancements and scientific discoveries (e.g., new biomarkers) in MS research. The analyses may include, but are not limited to, the following: genome-wide or candidate gene single nucleotide polymorphism analyses, candidate gene expression approaches, and the study of gene expression regulation. Additional possibilities will open up as the available data in MS research expand and associations of markers with MS disease progression or magnetic resonance imaging disease activities are demonstrated. A number of technically reliable technology platforms may be used to allow for a broad strategy for discovery and verification as well as potential integration of data to improve rationalization of markers that may be selected for follow-up validation.

14.2. Sample Size Considerations

This is an exploratory biobanking sub-study. No formal sample size or power calculations were conducted.

[5] The researchers/collaborators may engage third parties to conduct biomarker and biologic assessments.

CONFIDENTIAL
The information contained herein may not be used, disclosed, or published without the written consent of Biogen MA Inc.

15. ETHICAL REQUIREMENTS

Biogen and the Investigator must comply with all instructions, regulations, and agreements in this protocol and applicable International Council for Harmonisation (ICH) and Good Clinical Practice (GCP) guidelines and conduct the study according to local regulations.

The Investigator may delegate responsibilities for study-related tasks where appropriate to individuals sufficiently qualified by education, training, and experience, in accordance with applicable ICH and GCP guidelines. The Investigator should maintain a list of the appropriately qualified persons to whom significant study-related duties have been delegated.

15.1. Declaration of Helsinki

This sub-study will be performed in alignment with the ethical principles outlined in the Declaration of Helsinki.

15.2. Institutional Review Board/Ethics Committee

The Investigator must obtain IRB or ethics committee approval of the protocol, ICF, and other required study documents prior to starting the study. Biogen will submit documents on behalf of the investigational sites in countries other than the US in accordance with local practice and regulations.

If the Investigator makes any changes to the ICF, Biogen must approve the changes before the ICF is submitted to the IRB or ethics committee. A copy of the approved ICF must be provided to Biogen. After approval, the ICF must not be altered without the agreement of the relevant IRB or ethics committee and Biogen.

It is the responsibility of the Investigators to ensure that all aspects of institutional review are conducted in accordance with current applicable regulations.

Biogen must receive a letter documenting IRB or ethics committee approval, which specifically identifies the protocol, protocol number, and ICF, prior to the initiation of the study. Protocol amendments will be subject to the same requirements as the original protocol.

A progress report must be submitted to the IRB or ethics committee at required intervals and not less than annually.

At the completion or termination of the study, the investigational site must submit a close-out letter to the IRB or ethics committee and Biogen.

15.3. Patient Information and Consent

Prior to any data collection under this protocol, written informed consent with the approved ICF must be obtained from the patient or patient’s legally authorized representative (e.g., parent or legal guardian), as applicable, in accordance with local practice and regulations.

The background of the proposed study, the procedures, the benefits and risks of the study (including risks of the venipunctures/blood draws), and that study participation is voluntary for the patient must be explained to the patient (or the patient's legally authorized representative). The patient must be given sufficient time to consider whether to participate in the study.

Patients will be informed that their race and ethnicity will not be collected as part of this sub-study, but data collected in this sub-study may be linked to race and ethnicity information collected as part of Study 888MS001 for the purposes of future data analysis.

In addition, patients who have the capacity should provide their assent to participate in the study. The level of information provided to patients should match their level of understanding as determined by the Investigator and in accordance with applicable regulations and guidelines.

A copy of the signed and dated ICF (and assent, if applicable) must be given to the patient or the patient's legally authorized representative. The signed and dated ICF will be retained with the study records. Local regulations must be complied with in respect to the final disposition of the original (wet signature) and copies of the signed and dated ICFs.

Confirmation of informed consent (and assent, if applicable) must also be documented in the patient's medical record.

15.4. Patient Data Protection

Prior to any data collection or data sharing under this protocol, candidates must also provide all authorizations required by local law (e.g., PHI authorization in North America).

Patients' race and ethnicity will not be collected as part of this sub-study, but data collected in this sub-study may be linked to race and ethnicity information collected as part of Study 888MS001 for the purposes of future data analysis.

The patient will not be identified by name in the SRF or in any study reports, and these reports will be used for research purposes only. Biogen, its partners and designees, IRBs and ethics committees, and various government health agencies may inspect the records of this sub-study. Every effort will be made to keep the patient's personal medical data confidential.

15.5. Compensation for Injury

Biogen maintains appropriate insurance coverage for clinical studies and will follow applicable local compensation laws.

15.6. Conflict of Interest

The Investigators should address any potential conflicts of interest (e.g., financial interest in Biogen) with the patient before the patient makes a decision to participate in the study.

16. ADMINISTRATIVE PROCEDURES

16.1. Study Site Initiation

The Investigator must not enroll any patient in this sub-study or share any data prior to completion of a study initiation visit, conducted by Biogen or designee. This initiation visit will include a detailed review of the protocol and study procedures.

16.2. Quality Assurance

During and/or after completion of the study, quality assurance officers named by Biogen or the regulatory authorities may wish to perform onsite audits or inspections. The Investigator will be expected to cooperate with any audit or inspection and to provide assistance and documentation (including source data) as requested.

16.3. Monitoring of the Study

Biogen or its designee representatives may conduct onsite visits at the study facilities for the purpose of monitoring various aspects of the study. The Investigator must agree to Sponsor-authorized personnel having direct access to patient (or associated) files for the purpose of verifying entries made in any required study documentation and assist with their activities, if requested. Adequate space and time for monitoring visits should be made available by the Investigator or study staff. The site must complete any required study documentation in a timely manner and on an ongoing basis to allow regular review by the study team.

Monitoring visits must be conducted according to the applicable ICH and GCP guidelines to ensure protocol adherence, quality of data, compliance with regulatory requirements, and continued adequacy of the investigational site and its facilities.

16.4. Study Funding

Biogen is the Sponsor of the study and is funding the study. All financial details are provided in the separate contracts between the institution, Investigator, and Biogen.

16.5. Publications

Publications related to MS PATHS or using data within the LHS will be governed by the principles, rules, and processes developed by the MS PATHS steering committee.

CONFIDENTIAL
The information contained herein may not be used, disclosed, or published without the written consent of Biogen MA Inc.

17. FURTHER REQUIREMENTS AND GENERAL INFORMATION

17.1. External Contract Organizations

One or more central laboratory will be responsible for biosample management. Biogen will be responsible for all other aspects of this sub-study including but not limited to study initiation, monitoring, management of SAEs related to the venipunctures/blood draws, and data management.

17.1.1. Remote Data Capture

Data collected on the SRF will be entered by the central laboratory into their study data management system. The central laboratory will transfer the de-identified study data to Biogen, where it will be linked to additional de-identified data collected through MS PATHS studies (i.e., Study 888MS001 and future sub-studies) and made available for approved research.

17.2. Study Committee

A joint steering committee with membership from Biogen and participating health care institutions will be formed to provide strategic, scientific, and operational direction for MS PATHS Study 888MS001 and MS PATHS sub-studies, including this biobanking sub-study. The MS PATHS steering committee membership and charter will be included in the MS PATHS Program Guide. The membership and charter of any additional sub-committee will be formed (e.g., data use committee) and will also be included in the MS PATHS Program Guide.

17.3. Changes to Final Study Protocol

All protocol amendments must be submitted to the IRB or ethics committee and regulatory authorities if required by local law. Protocol modifications that affect patient safety, the scope of the investigation, or the scientific quality of the study must be approved by the IRB or ethics committee before implementation of such modifications to the conduct of the study. If required by local law, such modifications must also be approved by the appropriate regulatory agency prior to implementation.

However, Biogen may, at any time, amend this protocol to eliminate an apparent immediate hazard to a patient. In this case, the appropriate regulatory authorities will be notified subsequent to the modification.

In the event of a protocol modification, the ICF may require similar modifications (see Section 15).

CONFIDENTIAL
The information contained herein may not be used, disclosed, or published without the written consent of Biogen MA Inc.

17.4. IRB/Ethics Committee Notification of Study Completion or Termination

Where required, the regulatory authorities and IRBs or ethics committees must be notified of completion or termination of this sub-study, and sent a copy of the study synopsis in accordance with necessary timelines.

17.5. Retention of Study Data

The minimum retention time for study records will meet the strictest standard applicable to that site, as dictated by any institutional requirements or local laws or regulations. Prior to proceeding with destruction of records, the Investigator must notify Biogen in writing and receive written authorization from Biogen to destroy study records. In addition, the Investigator must notify Biogen of any changes in the archival arrangements including but not limited to archival at an offsite facility or transfer of ownership if the Investigator leaves the site.

CONFIDENTIAL
The information contained herein may not be used, disclosed, or published without the written consent of Biogen MA Inc.

18. REFERENCES

Achiron A, Feldman A, Magalashvili D, et al. Suppressed RNA-polymerase 1 pathway is associated with benign multiple sclerosis. PLoS One. 2012;7(10):e46871.

Confavreux C, Vukusic S, Moreau T, et al. Relapses and progression of disability in multiple sclerosis. N Engl J Med. 2000;343(20):1430-8.

De Jager PL. Genome-wide association study of severity in multiple sclerosis. Genes Immun. 2011;12(8):615-625.

Hecker M, Paap BK, Goertsches RH, et al. Reassessment of blood gene expression markers for the prognosis of relapsing-remitting multiple sclerosis. PLoS One. 2011;6(12):e29648.

IOM IoM. Best care at lower cost: The path to continuously learning health care in America. Washington, DC: The National Academies Press; 2013.

Martinelli-Boneschi F, Esposito F, Brambilla P, et al. A genome-wide association study in progressive multiple sclerosis. Mult Scler. 2012;18(10):1384-94.

Ottoboni L, Keenan BT, Tamayo P, et al. An RNA profile identifies two subsets of multiple sclerosis patients differing in disease activity. Sci Transl Med. 2012;4(153):153ra131.

Vecchio D, Naldi P, Collimedaglia L, et al. HLA-A*02 predicts severity, and duration of first multiple sclerosis attacks. Presented at the 5th Joint triennial congress of the European and Americas Committees for Treatment and Research in Multiple Sclerosis; 20 Oct 2011; Amsterdam, The Netherlands.

CONFIDENTIAL
The information contained herein may not be used, disclosed, or published without the written consent of Biogen MA Inc.

19. SIGNED AGREEMENT OF THE STUDY PROTOCOL

I have read the foregoing protocol, “Multiple Sclerosis Partners Advancing Technology and Health Solutions (MS PATHS) Biobanking Sub-Study for Future Biomarker and Biologic Research” and agree to conduct the study according to the protocol and the applicable ICH guidelines and GCP regulations, and to inform all who assist me in the conduct of this sub-study of their responsibilities and obligations.

____

Investigator’s Signature Date

____

Investigator’s Name (Print)

____

Study Site (Print)

CONFIDENTIAL
The information contained herein may not be used, disclosed, or published without the written consent of Biogen MA Inc.
