# Supplementary material for: Harnessing Real-World Data to Inform Decision-Making: Multiple Sclerosis Partners Advancing Technology and Health Solutions (MS PATHS)
Source: Front Neurol. 2020 Aug 7;11:632. doi: 10.3389/fneur.2020.00632 (PMC7426489; doi:10.3389/fneur.2020.00632)
Supplement: Supplementary file 3 [file Data_Sheet_3.PDF]

Biogen MA Inc.
250 Binney Street
Cambridge, MA 02142
United States

Biogen Idec Research Limited
Innovation House
70 Norden Road
Maidenhead Berkshire
SL6 4AY
United Kingdom

PROTOCOL NUMBER: 888MS001

PHASE OF DEVELOPMENT: Not applicable

PROTOCOL TITLE: Multiple Sclerosis Partners Advancing Technology and Health Solutions (MS PATHS) Demonstration Project

DATE: 03 April 2019
Version 2.0
FINAL

CONFIDENTIAL
The information contained herein may not be used, disclosed, or published without the written consent of Biogen MA Inc.

[redacted]

[redacted]

[redacted]

[redacted]

CONFIDENTIAL
The information contained herein may not be used, disclosed, or published without the written consent of Biogen MA Inc.

TABLE OF CONTENTS

1. SPONSOR INFORMATION 6
2. LIST OF ABBREVIATIONS 7
3. SYNOPSIS 8
4. SCHEDULE OF ACTIVITIES FOR PROJECT 888MS001 10
4.1. Schedule of Activities 10
5. INTRODUCTION 11
5.1. Overview of Multiple Sclerosis 11
5.2. Learning Health System 12
5.3. Project Rationale 13
6. PROJECT OBJECTIVE AND ENDPOINT 14
6.1. Objective 14
6.2. Endpoint 14
7. PROJECT DESIGN 15
7.1. Project Overview 15
7.2. Project Duration 16
7.3. Project Stopping Rules 16
7.4. End of Project 16
8. PROJECT POPULATION 17
8.1. Inclusion Criteria 17
8.2. Exclusion Criteria 17
9. ENROLLMENT AND REGISTRATION PROCEDURES 18
9.1. Screening and Enrollment 18
10. PROJECT PROCEDURES 19
11. WITHDRAWAL OF PATIENTS FROM PROJECT 20
12. SHARED DATA ELEMENTS OF ROUTINE CARE 21
12.1. Demographics and Socioeconomic Information 21
12.2. Multiple Sclerosis and Medical Information 21
12.3. Patient-Reported Outcome Information 22

12.4. Magnetic Resonance Imaging Information 22
13. SAFETY DEFINITIONS, RECORDING, REPORTING, AND RESPONSIBILITIES 24
13.1. Definitions 24
13.1.1. Adverse Event 24
13.1.2. Serious Adverse Event 24
13.2. Monitoring and Recording Events 25
13.2.1. Adverse Events 25
13.2.2. Serious Adverse Events 25
13.3. Prescribing Physician Responsibilities 25
13.4. Biogen Responsibilities 25
14. STATISTICAL METHODS AND DETERMINATION OF SAMPLE SIZE 26
15. ETHICAL REQUIREMENTS 27
15.1. Declaration of Helsinki 27
15.2. Ethics Committee 27
15.3. Patient Information and Consent 27
15.4. Patient Data Protection 28
15.5. Compensation for Injury 28
15.6. Conflict of Interest 28
16. ADMINISTRATIVE PROCEDURES 29
16.1. Project Site Initiation 29
16.2. Quality Assurance 29
16.3. Monitoring of the Project 29
16.4. Project Funding 29
16.5. Publications 29
17. FURTHER REQUIREMENTS AND GENERAL INFORMATION 30
17.1. Project Committees 30
17.2. Changes to Final Project Protocol 30
17.3. Ethics Committee Notification of Project Completion or Termination 30
17.4. Retention of Project Data 30
18. REFERENCES 31
19. SIGNED AGREEMENT OF THE PROJECT PROTOCOL 32

CONFIDENTIAL
The information contained herein may not be used, disclosed, or published without the written consent of Biogen MA Inc.

LIST OF TABLES

Table 1: Schedule of Activities..10

1. SPONSOR INFORMATION

In North America, Biogen MA Inc. (Cambridge, US) is the Sponsor of the project. In the Rest of World, Biogen Idec Research Limited (Maidenhead, UK) is the Sponsor of the project. Biogen is responsible for conducting the project.

Biogen MA Inc.<br>250 Binney Street<br>Cambridge, MA 02142<br>United States	Biogen Idec Research Limited<br>Innovation House<br>70 Norden Road<br>Maidenhead, Berkshire<br>SL6 4AY<br>United Kingdom

Biogen may transfer any or all of its project-related responsibilities to a contract research organization (CRO) and other third parties; however, Biogen retains overall accountability for these activities.

CONFIDENTIAL
The information contained herein may not be used, disclosed, or published without the written consent of Biogen MA Inc.

2. LIST OF ABBREVIATIONS

3D FLAIR	three-dimensional fluid-attenuated inversion recovery
3D MP RAGE	three-dimensional magnetization-prepared rapid gradient-echo imaging
AE	adverse event
EMR	electronic medical record
GVP	Good Pharmacovigilance Practices
ICF	informed consent form
ICH	International Conference on Harmonisation
IOM	Institute of Medicine
IRB	institutional review board
LHS	Learning Health System
MRI	magnetic resonance imaging
MS	multiple sclerosis
MS PATHS	Multiple Sclerosis Partners Advancing Technology and Health Solutions
MSPT	Multiple Sclerosis Performance Test
Neuro-QoL	Quality of Life Outcomes in Neurological Disorders
PHI	protected health information
RRMS	relapsing-remitting multiple sclerosis
SAE	serious adverse event
SPMS	secondary progressive multiple sclerosis

CONFIDENTIAL
The information contained herein may not be used, disclosed, or published without the written consent of Biogen MA Inc.

3. SYNOPSIS

Protocol Number:	888MS001
Protocol Title:	Multiple Sclerosis Partners Advancing Technology and Health Solutions (MS PATHS) Demonstration Project
Version Number:	1
Name of Project Treatment:	None
Project Indication:	Multiple sclerosis (MS)
Project Rationale:	Multiple Sclerosis Partners Advancing Technology and Health Solutions (MS PATHS) is a demonstration project that will collect data from standard-of-care activities across multiple MS centers in a standardized format. The goal of this real-world data collection is to better understand MS, to identify predictors of therapeutic responses, and to develop approaches to personalized medicine.
Phase of Development:	Not applicable
Project Objectives and Endpoints:	The objective of this demonstration project is to establish a database of de-identified standardized data collected as part of routine care that can be shared across collaborators for research purposes.<br><br>The endpoint of this demonstration project is the number of patients from each institution who have contributed de-identified data to the Learning Health System (LHS).
Project Design:	Patients with MS will be evaluated during their visit through each institution's routine care pathways, including the Multiple Sclerosis Performance Test (MSPT) and a magnetic resonance imaging protocol with common acquisition sequences (i.e., three-dimensional magnetization-prepared rapid gradient-echo imaging and three-dimensional fluid-attenuated inversion recovery). Clinicians will have access to all data for individual patients in their normal clinical data systems as part of their routine

	care practices.<br><br>This project will include patients with a confirmed diagnosis of clinically isolated syndrome or MS.<br><br>The MS PATHS demonstration project will provide an infrastructure to abstract and share select data collected from routine clinical practice directly from data collection tools (e.g., MSPT) and clinical data systems (e.g., electronic medical records). De-identified data abstracted from each collaborating institution will be stored in a common cloud-based data repository termed as the LHS.<br><br>The MS PATHS infrastructure will also provide a mechanism to create a register of patients who are willing to be contacted by local site personnel about future research studies to collect data outside of routine care (i.e., sub-studies). Sub-studies will fully leverage the data stored in the LHS and collect only the additional data needed for the sub-study.
Project Location:	Approximately 10 sites in the United States and Europe are planned.
Number of Planned Patients:	Approximately 9000 to 25,000 patients are planned to be included.
Project Population:	This project will be conducted in patients with MS or clinically isolated syndrome (henceforth referred to as MS patients).
Treatment Groups:	Not applicable
Duration of Project Participation	The demonstration project will last for approximately 6 years.

CONFIDENTIAL
The information contained herein may not be used, disclosed, or published without the written consent of Biogen MA Inc.

4. SCHEDULE OF ACTIVITIES FOR PROJECT 888MS001

4.1. Schedule of Activities

Table 1 outlines the Multiple Sclerosis Partners Advancing Technology and Health Solutions (MS PATHS)-specific activities and routine care data elements shared as part of MS PATHS.

Table 1: Schedule of Activities

**MS PATHS-Specific Activities**
MS PATHS consent (as determined by local institutional review board/ethics committee)
MS PATHS registration
**Routine Care Data Elements Shared (if Collected)**
Demographics
Employment status
Type of insurance coverage
Social support for MS-related problems
MS disease history
MS disease-modifying therapy usage
Other medication usage
Multiple Sclerosis Performance Test (iPad-based administration of processing speed test, manual dexterity test, low-contrast visual acuity, and Timed 25-Foot Walk)
MS relapses
Neuro-QoL (including sub-domains)
Patient-Determined Disease Steps
Mobility aid usage
MS-related laboratory test values
Anthropometric measurements and vital signs
Substance use (e.g., alcohol use and smoking status)
MRI DICOM for MS-related scans
Assessment of new or enlarging T2 lesions
Assessment of brain atrophy

DICOM = Digital Imaging and Communications in Medicine; MRI = magnetic resonance imaging; MS = multiple sclerosis; MS PATHS = Multiple Sclerosis Partners Advancing Technology and Health Solutions; Neuro-QoL = Quality of Life Outcomes in Neurological Disorders.

CONFIDENTIAL
The information contained herein may not be used, disclosed, or published without the written consent of Biogen MA Inc.

5. INTRODUCTION

5.1. Overview of Multiple Sclerosis

Multiple sclerosis (MS) is a chronic inflammatory demyelinating disease of the central nervous system that affects approximately 400,000 persons in North America and 365,000 persons in Europe. It is predominantly a disease of young adults, primarily women, with disease onset typically occurring between the ages of 20 and 40 years. MS is the most common form of inflammatory demyelinating central nervous system disease and the most common cause of nontraumatic neurological disability in young adults.

The most common form of MS is relapsing-remitting multiple sclerosis (RRMS), affecting approximately 58% of the overall MS population [Rodriguez 1994]. In most cases, patients with RRMS experience discrete episodes of neurologic dysfunction (referred to as relapses, exacerbations, or attacks), each lasting several days to several weeks, which occur at irregular intervals over many years. Symptoms of such relapses include loss of vision or double vision, numbness or tingling sensation in the extremities, muscle weakness, slurred speech, difficulty with coordination, and bladder dysfunction. Early in the course of this phase of the disease, these symptoms tend to subside completely after each attack. Over time, recovery from attacks may become incomplete, leading to the accumulation of functional disability.

Disease progression and advancing disability are common in patients with MS who are followed for several years. This process can begin insidiously from the onset of the disease (primary progression) or after 1 or more clinical flares (secondary progression). The majority of patients who start with RRMS eventually develop secondary progressive multiple sclerosis (SPMS). The precise date of onset of secondary progression is difficult to define; it is usually assessed retrospectively based on a pattern of continuous neurological worsening over at least 12 months [Polman 2005]. Cross-sectional epidemiological studies from large MS centers in North America (London, Ontario, and Canada) [Weinshenker 1998; Weinshenker 1989] and Europe (Lyon, France) [Confavreux and Vukusic 2006; Confavreux 2000] have shown that 30% to 40% of patients with MS at any given time suffer from progressive disease. In the cohort from Lyon, 11% had progression preceded by relapses that continue to occur, referred to as relapsing SPMS [Confavreux and Vukusic 2006; Confavreux 2000]; 16% had progression preceded by relapses that had ceased to occur or became infrequent, SPMS, or without superimposed relapses; and 15% experienced progression from onset, 9% without documented relapses (primary progressive MS) and 6% with at least 1 documented relapse (progressive-relapsing MS).

CONFIDENTIAL
The information contained herein may not be used, disclosed, or published without the written consent of Biogen MA Inc.

5.2. Learning Health System

The Institute of Medicine (IOM) issued a report in 2013 describing the concept of a Learning Health System (LHS) that capitalizes on the existing patient data that flow through the health care system to advance clinical knowledge with every patient visit [Institute of Medicine of the National Academies 2013]. The report outlined the necessity to evolve to such a system to achieve greater value in health care for all stakeholders. The American health care system has been struggling to achieve a level of consistent quality, efficiency, and safety across the nation that, if achieved, could save many lives. Paradoxes in the health care system have held back this advancement of care and value for a multitude of reasons. Although the IOM report is focused on the American health system, many health systems around the globe face similar challenges. First, the coexistence of over-treatment and under-treatment can leave patients with either unnecessary and expensive care or a lack of adequate care due to an absence of standard evidence-based protocols. Second, patients' health challenges and the health care system are becoming increasingly complex. As the population grows older, patients are developing more comorbid diseases, but treatment guidelines have not adapted to handle this challenge. Third, the American health system's understanding of diseases is expanding, but with the expansion of medical knowledge comes increasingly complex disease sub-types. Finally, new drugs and devices are being increasingly introduced, but evidence to support identifying the most effective drugs for various disease sub-types continues to lag behind. To move health care forward and establish evidence-based guidelines, an LHS needs to be formed.

The crux of an LHS is aggregation of data from routine clinical practice that can be subsequently used to generate knowledge and insights to improve patient care. The IOM lays out 4 important steps to achieving this LHS. First is generating and using real-time knowledge to improve outcomes. The second key step to achieving an LHS is creating a "culture of care" that supports the vision of continuous improvement with each patient visit. Physicians face pressures from time, chaotic environments, inefficient workflows, administrative burdens, and uncoordinated systems that can distract initiatives that focus on improving care. Having strong leadership and governance that incentivize systematic problem solving, experimentation, and learning from past experiences are essential to minimizing these distractions and focusing on advancement of clinical knowledge. The third step is engaging families, patients, and communities to improve communication and create a truly patient-centered care system. Engaging patients in their own care decisions allows them to give a personal perspective on the suitability of treatments, which can ultimately result in more effective care for the patient. Additionally, involving the patient by collecting self-reported outcomes can help engage them and expand the knowledge base without adding burden to the clinician. Finally, the fourth step is achieving and then rewarding high-value care through payment models that focus on value instead of individual services and products. Transparency in payment will help patients choose appropriate providers, which will, in turn, encourage providers and organizations to achieve higher value. These 4 steps can help create a true LHS, where clinicians, researchers, and, ultimately, patients will benefit from a focus on learning through data.

The MS population would especially benefit from such a system, as treatment protocols for MS are less standardized in comparison to other disease states, and few evidence-based treatment

guidelines exist to indicate which patients will respond best to the numerous therapies on the market.

5.3. Project Rationale

MS PATHS is a demonstration project of an LHS in MS. MS PATHS is being launched by Biogen in collaboration with select health care institutions.

This demonstration project will specifically focus on leveraging technology to generate and aggregate de-identified data from routine care visits across multiple institutions. The goal is to ultimately improve outcomes in MS by enabling real-world, broad-scale MS phenotyping that is needed to better understand MS, predict therapeutic responses, and develop meaningful approaches to personalized medicine.

MS PATHS aims to engage all providers and nearly all MS patients in an MS center to standardize, quantify, and maximize data collected as part of routine care. The demonstration project plans to leverage existing and developing technology with the goal of reducing the data collection burden on health care providers and staff (e.g., Multiple Sclerosis Performance Test [MSPT]). In addition, the demonstration project anticipates launching separately consented sub-studies to collect data outside of routine care. This infrastructure aims to enable researchers to access a large set of longitudinal patient data to advance the knowledge of MS disease progression and patients' response to therapies.

CONFIDENTIAL
The information contained herein may not be used, disclosed, or published without the written consent of Biogen MA Inc.

6. PROJECT OBJECTIVE AND ENDPOINT

6.1. Objective

The objective of this demonstration project is to establish a database of de-identified standardized data collected as part of routine care that can be shared across collaborators for research purposes.

6.2. Endpoint

The ability to establish a database of de-identified standardized data for research purposes will be evaluated by the number of patients from each institution who have contributed to the LHS.

7. PROJECT DESIGN

7.1. Project Overview

Participating health care institutions will have MS centers that use the MSPT (including a Quality of Life Outcomes in Neurological Disorders [Neuro-QoL] and other patient-reported data modules) and a magnetic resonance imaging (MRI) protocol with common acquisition sequences (i.e., three-dimensional magnetization-prepared rapid gradient-echo imaging [3D MP RAGE] and three-dimensional fluid-attenuated inversion recovery [3D FLAIR]) as part of their routine care practice.

The MS PATHS demonstration project will include patients with a confirmed diagnosis of clinically isolated syndrome or MS (henceforth referred to as MS patients). Approximately 9000 to 25,000 patients are planned to be included in the project, and at approximately 10 sites in the United States and Europe.

MS patients will be evaluated during their visit through each institution's routine care pathways, including the MSPT and an MRI protocol with common acquisition sequences (i.e., 3D MP RAGE and 3D FLAIR), when applicable. Clinicians will have access to all data for individual patients in their normal clinical data systems as part of their routine care practice.

The MS PATHS demonstration project will provide an infrastructure to abstract and share select data collected from routine clinical practice.[1] De-identified data abstracted from each collaborating institution will be stored in a common cloud-based data repository termed as the LHS.[2] The MS patient (or MS patient's legal representative; see Section 15.3) will provide any required informed consent (or assent) and authorization to use protected health information (PHI) in accordance with national and local subject privacy regulations. Access to the LHS will be governed by the principles, rules, and processes developed by the MS PATHS steering committee.

The MS PATHS infrastructure will also provide a mechanism to create a register of patients who are willing to be contacted by local site personnel about future research studies to collect data outside of routine care (i.e., sub-studies). Sub-studies will fully leverage the data stored in the LHS and collect only the additional data needed for the sub-study, with the goal of creating cost and time efficiencies. Throughout the demonstration project, local staff may contact patients within their institution who have given permission to be contacted for recruitment into appropriate sub-studies that have been approved by the local institutional review board (IRB). The patient (or patient's legal representative) will provide any required additional consent or assent prior to being enrolled in an MS PATHS sub-study.

[1] The planned infrastructure aims to enable data sharing directly from data collection tools (e.g., MSPT), clinical data systems (e.g., EMRs), and other applicable technology. Further information about information technology infrastructure will be available in a separate MS PATHS information technology infrastructure overview.
[2] Over time, the LHS may add additional functionality such as a cloud-based analytical environment.

CONFIDENTIAL
The information contained herein may not be used, disclosed, or published without the written consent of Biogen MA Inc.

7.2. Project Duration

The demonstration project will last for approximately 6 years. Once an MS PATHS Participant Identifier (hereafter referred to as the MS PATHS ID) is generated for the patient, data will be shared prospectively for the duration of the demonstration project or until a patient opts out and/or withdraws consent or assent. In addition, retrospective data will be shared for the 12-month period prior to an MS PATHS ID being generated for an MS patient.

7.3. Project Stopping Rules

The Sponsor may terminate this demonstration project if proper notice is given to the collaborating healthcare institutions, as stipulated in the project contracts.

7.4. End of Project

The end of this demonstration project is 6 years after the first MS patient among participating health care institutions has a routine care visit that utilizes both the MSPT and has an MRI scan that includes the common acquisition sequences.

8. PROJECT POPULATION

8.1. Inclusion Criteria

To be eligible to participate in this demonstration project, patients under the care of a physician at an MS center participating in MS PATHS must meet the following criteria:

1. Have a confirmed diagnosis of MS or clinically isolated syndrome.

2. Patient (or patient's legal representative) has the ability to understand the purpose and risks of the project and provide any required signed and dated informed consent form (ICF), or assent form, and authorization to use PHI in accordance with national and local subject privacy regulations.

8.2. Exclusion Criteria

Patients will be excluded from the demonstration project if any of the following exclusion criteria exist:

1. Patients under 18 years of age will be excluded unless their parent or legal guardian provides any required signed and dated ICF and authorization to use PHI and patients under 18 years of age provide assent (as needed) in accordance with national and local subject privacy regulations.

2. Other unspecified reasons that, in the opinion of the Investigator or Biogen, make the patient unsuitable for participation in the demonstration project. A patient only needs to complete the assessments deemed necessary by the Investigator.

CONFIDENTIAL
The information contained herein may not be used, disclosed, or published without the written consent of Biogen MA Inc.

9. ENROLLMENT AND REGISTRATION PROCEDURES

9.1. Screening and Enrollment

If written informed consent or assent is required for participation and/or data sharing, patients must be consented before any assessments requiring consent are performed. At the time of consent, the patient will be enrolled into the project.

CONFIDENTIAL
The information contained herein may not be used, disclosed, or published without the written consent of Biogen MA Inc.

10. PROJECT PROCEDURES

MS patients will be provided with information about the MS PATHS project prior to agreeing to participate. Informed consent or assent (if required) will be obtained after the patient receives the information about the demonstration project and has had the opportunity to ask questions about the project.

All MS patients who participate in the MS PATHS demonstration project will be assigned a unique MS PATHS ID, which acts as the patient identifier in the LHS, linking de-identified data from different sources for the same patient. The link between the patient's medical record number and the MS PATHS ID is permanently destroyed after the MS PATHS ID is generated.

Data will be shared from the patients' routine clinical visits for MS care prospectively, as well as any data from routine clinical care for MS during the 12 months prior to assignment of their MS PATHS ID. Routine clinical visits for MS care will encompass visits to outpatient MS specialty center or neurology clinics and associated MRI imaging studies and laboratory tests.

CONFIDENTIAL
The information contained herein may not be used, disclosed, or published without the written consent of Biogen MA Inc.

11. WITHDRAWAL OF PATIENTS FROM PROJECT

Patients will be withdrawn from the project if they are unable or unwilling to comply with project requirements that include the following:

- The patient (or patient's legal representative) opts out of data sharing or withdraws consent (or assent).
- Unspecified reasons that, in the opinion of the Investigator or Biogen, make the patient unsuitable for continued participation.

The date and reason for the patient's withdrawal from the project must be recorded in the patient's medical record.

CONFIDENTIAL
The information contained herein may not be used, disclosed, or published without the written consent of Biogen MA Inc.

12. SHARED DATA ELEMENTS OF ROUTINE CARE

Select de-identified patient data generated as part of routine MS clinical care will be shared in the LHS from clinical data collection tools (e.g., the MSPT) and hospital source systems (e.g., electronic medical record [EMR]).

Once an MS PATHS ID is generated for the patient, data will be shared prospectively for the duration of the demonstration project or until a patient (or patient's legal representative) opts out and/or withdraws consent (or assent). In addition, retrospective data will be shared for the 12-month period prior to an MS PATHS ID being generated for an MS patient.

12.1. Demographics and Socioeconomic Information

The following demographic and socioeconomic data elements will be shared, if collected:

- age
- gender
- race
- ethnicity
- employment status
- type of insurance coverage
- living situation
- social support for MS-related problems

12.2. Multiple Sclerosis and Medical Information

The following MS and medical data elements will be shared, if collected:

- MS disease history
- use of MS disease-modifying therapies[3]
- use of other medications[4]

[3] Start and stop dates will be included in the LHS.
[4] Start and stop dates will be included in the LHS.

CONFIDENTIAL
The information contained herein may not be used, disclosed, or published without the written consent of Biogen MA Inc.

- MSPT results (i.e., iPad-based administration of processing speed test, manual dexterity test, low-contrast visual acuity, and Timed 25-Foot Walk)
- MS relapses
- mobility aid usage[5]
- MS-related laboratory test values (e.g., Vitamin D levels, John Cunningham virus anti-body test)[6]
- anthropometric measurements and vital signs
- substance use (e.g., alcohol use and smoking status)

12.3. Patient-Reported Outcome Information

The following patient-reported outcome data elements will be shared, if collected:

- Patient-Determined Disease Steps
- Neuro-QoL (including sub-domains), as follows:
  - physical – function/health
  - physical – symptoms
  - mental – emotional health
  - mental – cognitive health
  - social

12.4. Magnetic Resonance Imaging Information

The following MRI data elements will be shared, if collected:

- Digital Imaging and Communications in Medicine data for MS-related scans (i.e., 3D MP RAGE and 3D FLAIR)
- semi-quantitative assessment of brain atrophy (if available)
- semi-quantitative assessment of new or enhancing T2 lesions (if available)

[5] Start and stop dates will be included in the LHS.
[6] Results of sensitive laboratory tests such as sexually transmitted diseases will not be included in the LHS.

CONFIDENTIAL
The information contained herein may not be used, disclosed, or published without the written consent of Biogen MA Inc.

- quantitative assessment of brain atrophy (if available)
- quantitative assessment of new or enhancing T2 lesions (if available)

CONFIDENTIAL
The information contained herein may not be used, disclosed, or published without the written consent of
Biogen MA Inc.

13. SAFETY DEFINITIONS, RECORDING, REPORTING, AND RESPONSIBILITIES

Adverse event (AE) reporting in the form of individual case safety reports is not required for noninterventional post-authorization studies based on secondary use of data. AEs captured as endpoint(s) defined in the project protocol will be summarized in the final project report and in any interim safety analysis, if applicable.

13.1. Definitions

13.1.1. Adverse Event

An AE is any undesirable experience associated with the use of a medical product in a patient. An AE can therefore be any unfavorable and unintended sign, symptom, or disease temporally associated with the use of a medicinal product, whether or not related to the medicinal product. A pre-existing condition that worsens in severity would also be considered an AE.

13.1.2. Serious Adverse Event

A serious adverse event (SAE) is any untoward medical occurrence that at any dose:

- Results in death
- In the view of the Prescribing Physician, places the patient at immediate risk of death (a life-threatening event); however, this does not include an event that, had it occurred in a more severe form, might have caused death
- Requires inpatient hospitalization or prolongation of existing hospitalization
- Results in persistent or significant disability/incapacity
- Results in a congenital anomaly/birth defect

An SAE may also be any other medically important event that, in the opinion of the Prescribing Physician, may jeopardize the patient or may require intervention to prevent 1 of the other outcomes listed in the definition above. Examples of such medical events include allergic bronchospasm requiring intensive treatment in an emergency room or convulsions occurring at home that do not require an inpatient hospitalization. While MS relapses resulting in hospitalization are considered SAEs, they are not typically reported in non-interventional post-authorization studies unless, in the opinion of the Prescribing Physician, a relapse is complicated by other SAEs.

CONFIDENTIAL
The information contained herein may not be used, disclosed, or published without the written consent of Biogen MA Inc.

13.2. Monitoring and Recording Events

13.2.1. Adverse Events

AEs will not be collected as part of this project. Any AEs of which a participating Prescribing Physician becomes aware of should follow spontaneous postmarketing rules as per local regulations.

13.2.2. Serious Adverse Events

SAEs will not be collected as part of this project. Any SAEs of which a participating Prescribing Physician becomes aware of should follow spontaneous postmarketing rules as per local regulations.

13.3. Prescribing Physician Responsibilities

The Prescribing Physician's responsibilities include the following: the reporting of AEs and SAEs following spontaneous postmarketing rules as per local regulations.

13.4. Biogen Responsibilities

Biogen's responsibilities include the report of AEs and SAEs received for Biogen products as per local regulations.

14. STATISTICAL METHODS AND DETERMINATION OF SAMPLE SIZE

The data collected as part of routine care and shared as part of this project will be available in de-identified form to the Sponsors and researchers from participating MS PATHS institutions for a variety of research questions and statistical analyses that are not yet determined. Use of data for specific research questions not explicitly stated in the current protocol will be described by separate project protocols and statistical analysis plans. A few examples of potential areas of scientific and statistical exploration are provided below for illustrative purposes:

- Descriptive characterization of the MS PATHS patient population in terms of demographics, MS history, MRI, MSPT measures, and health-related quality of life, as measured by Neuro-QoL.
- Assessment of patient characteristics that are predictive of changes in MSPT measures, MRI outcomes, and Neuro-QoL over time.
- Assessment of the relationship between MSPT measures, MRI outcomes, and Neuro-QoL over time.
- Assessment of patient characteristics and clinical measures that are associated with treatment outcomes.

Data shared under this project may continue to be used for research purposes by Biogen, those working on behalf of Biogen and affiliates of Biogen, and Biogen's Research Collaborators for research purposes after the demonstration project ends.

This project is an exploratory demonstration project. There is no predetermined sample size, as this will be dependent on the number of patients participating across MS centers at participating health care institutions. No formal sample size or power calculations have been conducted.

CONFIDENTIAL
The information contained herein may not be used, disclosed, or published without the written consent of Biogen MA Inc.

15. ETHICAL REQUIREMENTS

Biogen and the Investigator must comply with all instructions, regulations, and agreements in this protocol and applicable International Conference on Harmonisation (ICH) and Good Pharmacovigilance Practices (GVP) guidelines and conduct the project according to local regulations.

15.1. Declaration of Helsinki

This study will be performed in alignment with the ethical principles outlined in the Declaration of Helsinki.

15.2. Ethics Committee

The Investigator must obtain IRB or ethics committee approval of the protocol or notice of exemption, ICF (if applicable), and other required project documents prior to starting the project.

If the Investigator makes any changes to the ICF (if applicable), Biogen must approve the changes before the ICF is submitted to the IRB or ethics committee. A copy of the approved ICF must be provided to Biogen. After approval, the ICF must not be altered without the agreement of the relevant IRB or ethics committee and Biogen.

It is the responsibility of the Investigator to ensure that all aspects of institutional review are conducted in accordance with current governmental regulations.

Biogen must receive a letter documenting IRB or ethics committee approval or notice of exemption, which specifically identifies the protocol, protocol number, and ICF (if applicable) prior to the initiation of the project. Protocol amendments will be subject to the same requirements as the original protocol.

A progress report must be submitted to the IRB or ethics committee at required intervals as determined by the IRB or ethics committee. Documentation of required submissions must be provided to Biogen unless exempt from IRB or ethics committee review.

At the completion or termination of the project, the participating site must submit a close-out letter to the IRB or ethics committee (if applicable) and Biogen.

15.3. Patient Information and Consent

If required by local IRB, ethics committee, or other oversight body, prior to any data collection and data sharing under this protocol, written informed consent or assent with the approved ICF must be obtained from the patient or patient's legally authorized representative, as applicable, in accordance with local practice and regulations.

CONFIDENTIAL
The information contained herein may not be used, disclosed, or published without the written consent of Biogen MA Inc.

If informed consent (or assent) is required, information about the project and that project participation is voluntary must be explained to the patient. The patient must be given sufficient time to consider whether to participate in the project. A copy of the ICF, signed and dated by the patient, must be given to the patient. Confirmation of a patient's informed consent or assent must also be documented in the patient's medical record prior to any data collection or data sharing under this protocol.

Each ICF should contain an authorization allowing the Investigator and Biogen to use and disclose PHI in compliance with local law.

The signed ICF will be retained with the project records.

15.4. Patient Data Protection

Prior to any data collection or data sharing under this protocol, patients must also provide all authorizations required by local law (e.g., PHI authorization in North America).

Identified patient data will not be accessible to Biogen. Patient data will be de-identified before it is made available in the LHS for research purposes.[7]

15.5. Compensation for Injury

Biogen maintains appropriate insurance coverage for clinical studies and will follow applicable local compensation laws.

15.6. Conflict of Interest

The Investigator should address any potential conflicts of interest (e.g., financial interest in Biogen) with the patient before the patient makes a decision to participate in the project.

[7] Further information about data de-identification will be available in a separate MS PATHS data privacy plan.

CONFIDENTIAL
The information contained herein may not be used, disclosed, or published without the written consent of Biogen MA Inc.

16. ADMINISTRATIVE PROCEDURES

16.1. Project Site Initiation

The Investigator must not enroll any patients or share any data in this project prior to completion of a project initiation visit conducted by Biogen or designee. This initiation visit will include a detailed review of the protocol and project procedures.

16.2. Quality Assurance

During and/or after completion of the project, quality assurance officers named by Biogen or the regulatory authorities may wish to perform onsite audits. The Investigator will be expected to cooperate with any audit and to provide assistance and documentation (including source data) as requested.

16.3. Monitoring of the Project

Biogen or its designee representatives may conduct onsite visits at the project facilities for the purpose of monitoring various aspects of the project. The Investigator must agree to Sponsor-authorized personnel having direct access to patient (or associated) files for the purpose of verifying entries made in any required project documentation and assist with their activities, if requested. Adequate space and time for monitoring visits should be made available by the Investigator or project staff. The site must complete any required project documentation in a timely manner and on an ongoing basis to allow regular review by the project team.

16.4. Project Funding

Biogen is the Sponsor of the project and is funding the project. All financial details are provided in separate contract(s) between the institution, Investigator, and Biogen.

16.5. Publications

Publications related to MS PATHS or using data within the LHS will be governed by the principles, rules, and processes developed by the MS PATHS steering committee.

CONFIDENTIAL
The information contained herein may not be used, disclosed, or published without the written consent of Biogen MA Inc.

17. FURTHER REQUIREMENTS AND GENERAL INFORMATION

17.1. Project Committees

A joint steering committee with membership from Biogen and participating health care institutions will be formed to provide strategic, scientific, and operational direction for the demonstration project. Steering committee membership and charter will be included in the MS PATHS Enablement Guide. The membership and charter of any additional sub-committee will be formed (e.g., data use committee) and will also be included in the MS PATHS Enablement Guide.

17.2. Changes to Final Project Protocol

Unless exempted from review, all protocol amendments must be submitted to the IRB or ethics committee and regulatory authorities, if required by local law. Unless exempted from review, protocol modifications that affect patient safety, the scope of the investigation, or the scientific quality of the project must be approved by the IRB or ethics committee before implementation of such modifications to the conduct of the project. If required by local law, such modifications must also be approved by the appropriate regulatory agency prior to implementation.

However, Biogen may, at any time, amend this protocol to eliminate an apparent immediate hazard to a patient. In this case, the appropriate regulatory authorities will be notified subsequent to the modification.

In the event of a protocol modification, the patient ICF (if applicable) may require similar modifications (see Sections 15.2 and 15.3).

17.3. Ethics Committee Notification of Project Completion or Termination

Where required, IRB or ethics committees must be notified of completion or termination of this project and sent a copy of the project synopsis in accordance with necessary timelines.

17.4. Retention of Project Data

The minimum retention time for project records will meet the strictest standard applicable to that site, as dictated by any institutional requirements or local laws or regulations. Prior to proceeding with destruction of records, the Investigator must notify Biogen in writing and receive written authorization from Biogen to destroy project records. In addition, the Investigator must notify Biogen of any changes in the archival arrangements, including, but not limited to, archival at an offsite facility or transfer of ownership if the Investigator leaves the site.

CONFIDENTIAL
The information contained herein may not be used, disclosed, or published without the written consent of Biogen MA Inc.

18. REFERENCES

Confavreux C, Vukusic S. Natural history of multiple sclerosis: a unifying concept. Brain. 2006;129(Pt 3):606-16. Epub 2006/01/18.

Confavreux C, Vukusic S, Moreau T, et al. Relapses and progression of disability in multiple sclerosis. N Engl J Med. 2000;343(20):1430-8.

Institute of Medicine of the National Academies. Best Care at Lower Cost: The path to continuously learning health care in america. Washington DC: The National Academies Press; 2013.

Polman CH, Reingold SC, Edan G, et al. Diagnostic criteria for multiple sclerosis: 2005 revisions to the "McDonald Criteria". Ann Neurol. 2005;58(6):840-6. Epub 2005/11/12.

Rodriguez M, Siva A, Ward J, et al. Impairment, disability, and handicap in multiple sclerosis: a population-based study in Olmsted County, Minnesota. Neurology. 1994;44(1):28-33.

Weinshenker BG. The natural history of multiple sclerosis: update 1998. Semin Neurol. 1998;18(3):301-7.

Weinshenker BG, Bass B, Rice GP, et al. The natural history of multiple sclerosis: a geographically based study. I. Clinical course and disability. Brain. 1989;112(Pt 1):133-146.

CONFIDENTIAL
The information contained herein may not be used, disclosed, or published without the written consent of Biogen MA Inc.

19. SIGNED AGREEMENT OF THE PROJECT PROTOCOL

I have read the foregoing protocol, "Multiple Sclerosis Partners Advancing Technology and Health Solutions (MS PATHS) Demonstration Project" and agree to conduct the project according to the protocol and the applicable ICH guidelines and GVP regulations, and to inform all who assist me in the conduct of this project of their responsibilities and obligations.

____

Investigator's Signature Date

____

Investigator's Name (Print)

____

Project Site (Print)

CONFIDENTIAL
The information contained herein may not be used, disclosed, or published without the written consent of Biogen MA Inc.
